# Single-cell analysis identifies cellular markers of the HIV permissive cell

**DOI:** 10.1371/journal.ppat.1006678

**Published:** 2017-10-26

**Authors:** Sylvie Rato, Antonio Rausell, Miguel Muñoz, Amalio Telenti, Angela Ciuffi

**Affiliations:** 1 Institute of Microbiology, Lausanne University Hospital and University of Lausanne, Lausanne, Switzerland; 2 INSERM UMR 1163, Imagine Institute, Paris, France; 3 Paris Descartes University–Sorbonne Paris Cité, Imagine Institute, Clinical Bioinformatics Lab, Paris, France; 4 The J. Craig Venter Institute, La Jolla, California, United States of America; Vaccine Research Center, UNITED STATES

## Abstract

Cellular permissiveness to HIV infection is highly heterogeneous across individuals. Heterogeneity is also found across CD4^+^ T cells from the same individual, where only a fraction of cells gets infected. To explore the basis of permissiveness, we performed single-cell RNA-seq analysis of non-infected CD4^+^ T cells from high and low permissive individuals. Transcriptional heterogeneity translated in a continuum of cell states, driven by T-cell receptor-mediated cell activation and was strongly linked to permissiveness. Proteins expressed at the cell surface and displaying the highest correlation with T cell activation were tested as biomarkers of cellular permissiveness to HIV. FACS sorting using antibodies against several biomarkers of permissiveness led to an increase of HIV cellular infection rates. Top candidate biomarkers included CD25, a canonical activation marker. The combination of CD25 high expression with other candidate biomarkers led to the identification of CD298, CD63 and CD317 as the best biomarkers for permissiveness. CD25^high^CD298^high^CD63^high^CD317^high^ cell population showed an enrichment of HIV-infection of up to 28 fold as compared to the unsorted cell population. The purified hyper-permissive cell subpopulation was characterized by a downregulation of interferon-induced genes and several known restriction factors. Single-cell RNA-seq analysis coupled with functional characterization of cell biomarkers provides signatures of the “HIV-permissive cell”.

## Author summary

CD4^+^ T cells are the main target of human immunodeficiency virus (HIV) infection. However, CD4^+^ T cells are not equally permissive to infection, varying between individuals and across cells isolated from the same individual. We explored cellular heterogeneity by analyzing the transcriptome profile of CD4^+^ T cells at single-cell level. Results identified T-cell receptor-mediated activation as the major determinant of CD4^+^ T cell heterogeneity. We identified cell surface proteins that highly correlated with T cell activation and HIV permissiveness. Activated CD4^+^ T cells expressing CD25, CD298, CD63 and CD317 were highly enriched for HIV permissiveness. The single-cell analysis approach used in this study allowed for the identification of the most HIV-permissive cell.

## Introduction

*In vivo* and *in vitro* data indicates that only a small fraction of the CD4^+^ T cell population is successfully infected by HIV. Cellular permissiveness to HIV infection differs between cell lines originating from different tissues, between T cell lines [1], between primary CD4^+^ T cells isolated from different HIV-negative blood donors [2], as well as between primary CD4^+^ T cells from the same donor [3]. HIV permissiveness in primary CD4^+^ T cells has been linked to: (i) activation, *i*.*e*. proliferating activated CD4^+^ T lymphocytes are more susceptible to HIV infection compared to resting CD4^+^ T cells [4–8]; (ii) specific CD4^+^ T cells subsets, such as effector memory cells [8, 9] or specifically, CCR4^+^CCR6^+^ and CXCR3^+^CCR6^+^ CD4^+^ T cells [10]; and (iii) expression of specific HIV dependent/restriction cellular factors that can modulate HIV replication [3]. Differences in permissiveness in human cell lines was recently associated with transcriptional and functional defects in key components of innate immunity [1]. Nevertheless, the specific phenotypic and functional characteristics of primary CD4^+^ T cells that are permissive to HIV infection remain elusive.

Single-cell technology is a valuable tool to study cellular heterogeneity in different biological settings, including virology [11–13]. Single-cell sequencing permits to explore whether inter-individual differences in successful infection can result from within-individual heterogeneity across individual cells. Cellular heterogeneity within an individual can arise from the presence of different subsets of CD4^+^ T cells [14, 15], differences in response to TCR activation [16], or other determinants such as the expression of innate immunity genes [17–20]. This suggests a model where a highly permissive individual possesses CD4^+^ T cells that are enriched in a given cell lineage, activate more rapidly or differently, express less antiviral factors, or a combination of factors.

Heterogeneity at the cellular level can result from differences in cell fate, i.e. permanent and irreversible commitment to a lineage, or in cell state, resulting from transient and reversible processes. Cell fate heterogeneity can lead to a mixture of cells of different types. Cell state differences arise from the intermediate stages of differentiation or activation of a cell type, or from stochasticity of gene expression, cell cycle, pulsating expression, circadian rhythms or level of reactivity to stimuli [21]. There is also increasing attention to the possibility of monoallelic expression, determining the use of different haplotypes in a given moment [22, 23]. Microfluidic control of cell capture and preparation of RNA-seq libraries from single cells allows the study of transcriptional heterogeneity in a reliable way. This methodology was successfully used to identify subpopulations and identify markers from many cell types [24–26].

The aim of this study was to use a *non a priori* and single-cell approach to identify molecular features that characterize the CD4^+^ T cell population most permissive to HIV infection. We hypothesized that permissiveness could be a feature of a specific cell lineage(s) (cell fate), or a feature of cells in a particular state. To this end, we performed comparative single-cell RNA-seq analyses and large-scale immunoprofiling on non-infected CD4^+^ T cells from uninfected individuals with extreme phenotypes of susceptibility to *in vitro* HIV infection. We showed that cellular activation state (degree of response to TCR stimulation) was the main factor of transcriptional heterogeneity at single cell level, which in turn translated in varying degrees of HIV permissiveness. Importantly, the permissive cell was identified before infection, thus revealing the biological basis of baseline CD4^+^ T cell susceptibility to HIV. Moreover, this single-cell based approach allowed the identification of specific biomarkers that partition cell populations into high and low permissive subsets. The combination of multiple candidate biomarkers further selected for highly permissive cells, thus defining the “HIV-permissive cell”.

## Results

### Single-cell RNA-seq analysis of CD4^+^ T cells from high and low HIV permissiveness donors identifies T cell activation as the major driver of transcriptional heterogeneity

**Fig 1:**
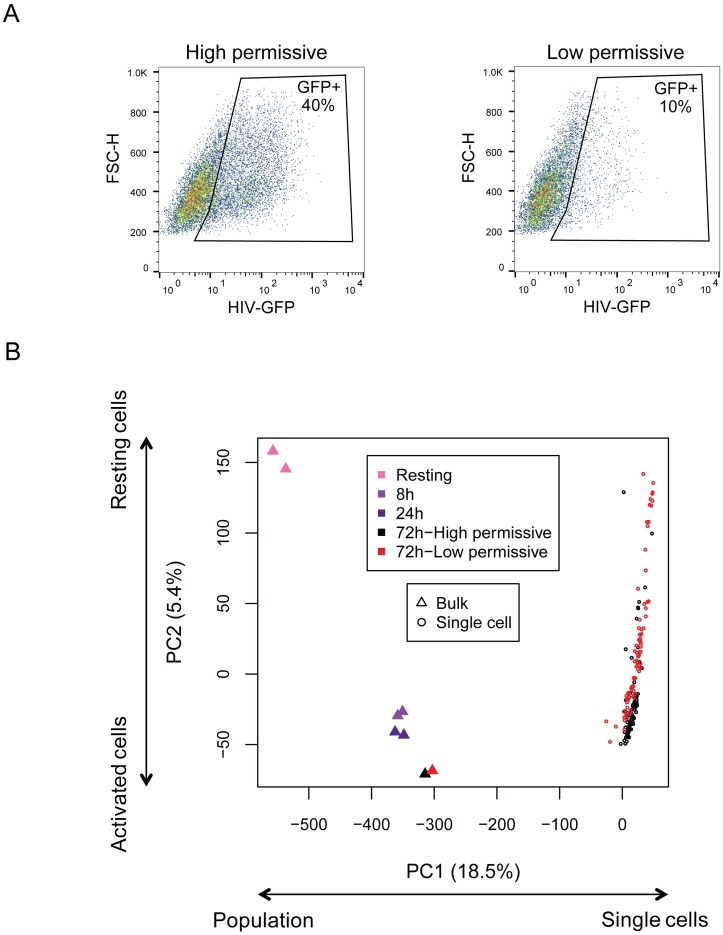
Single-cell transcriptome analysis of low and high permissive TCR-activated CD4^+^ T cells. **(A)** Susceptibility to HIV-GFP infection of cells isolated from a donor with high permissive cells (#42) and from a donor with low permissive cells (#123). FACS plot analysis shows forward scatter plot (FSC) on the y axis and GFP expression on the x axis. The region delineates GFP^+^ infected cells and the proportion of GFP^+^ cells is indicated. **(B)** Principal Component Analysis (PCA) of single-cells (dots) from the high permissive (black) and low permissive (red) donors as comparison to bulk populations (triangles). Bulk sequencing was performed from cells collected at resting (pink), and after TCR activation (8h, 24h and 72h post-activation, purple). PCA was performed on gene expression levels expressed as the log10 of the number of library size-normalized reads per kilobase of exonic sequence (Methods).

To explore the basis of permissiveness in primary CD4^+^ T cells at a single-cell level we took advantage of a previous study where a panel of donors was characterized for their heterogeneous permissiveness to HIV infection [3]. Cells from 18 donors were TCR-activated in presence of IL-2 for three days and evaluated for their cell growth and permissiveness using VSV-G-pseudotyped eGFP expressing HIV-based vector (named HIV-GFP, S1 Fig). Two donors, “42” and “123”, were selected as displaying different levels of permissiveness (high and low permissive with ~40% and ~10% GFP^+^ infected cells, respectively), while showing similar growth capacity (Fig 1A and S1 Fig).

To investigate cell heterogeneity without *a priori*, uninfected CD4^+^ T cells from the selected donors were used for single-cell RNA-seq analysis using Fluidigm C1 technology. This technology is based on a microfluidic device that uses pre-defined plates based on cell size to separate single cells. To isolate activated CD4^+^ T cells, we used the medium size chip that can accommodate cells ranging from 10 to 17 μm (± 2 μm). Although the use of pre-specified size might introduce a bias, it more likely minimizes the difference between the two donors. Population RNA sequencing was performed in parallel on bulk cell preparations. The transcriptomes of 85 and 81 individual cells from the high and from the low permissive donor, respectively, were profiled. An average of 25 million reads per cell was obtained. Of these, an average of 6.6 million (standard deviation 2.7 million) paired reads (fragments) per cell was uniquely mapped. Average gene expression levels across individual cells from the same donor showed high correlation with the expression levels assessed on the equivalent bulk cell samples sequenced by population RNA-seq (r = 0.78 and 0.77 for the high and low permissive donor, respectively). Such high correlations served as a quality control of the overall single-cell sequencing procedure. The pairwise comparisons among all individual cells from the same individual showed a distribution of Spearman rank correlations of gene expression levels with median of 0.72 and 0.58 for the high and low permissive donor, respectively, indicating that transcriptional profiles of individual cells from the low permissive donor were more heterogeneous than those of the high permissive donor (Wilcoxon-rank sum test p-value < 2.2e-16; S2 Fig).

We then explored transcriptional heterogeneity by *Sincell* software for the statistical assessment of cell-state hierarchies developed in our laboratory [27]. The analysis showed a continuum of transcriptional cell state for both donors, without branching patterns (S3 Fig). This continuum was also observed in the Principal Component Analysis (PCA) of the single-cell libraries from the donors, together with RNA-seq libraries from reference bulk cell samples (resting CD4^+^ T cells and activated CD4^+^ T cells at 8h, 24h and 72h after TCR stimulation) (Fig 1B). The two main principal axes–PC1 and PC2- explained 18.5% and 5.4% of the total variance, respectively. PC1 presumably gathered heterogeneity related to the experimental protocol (single-cell *versus* bulk sequencing). PC2 ordered bulk cell samples most likely according to activation state, following time after TCR-mediated activation (resting, 8h, 24h and 72h), and supported by Pearson correlation with the prototypical activation markers CD25 (r = 0.562 with PC2, while r = 0.089 with PC1). Equivalent PCA analysis of the single-cell transcriptomes without considering the bulk samples confirmed this result (S4 Fig), with the main principal axis of single individual cells (PC1, S4A Fig) highly correlating with PC2 axis of bulk population (S4B Fig, Pearson r = 0.9994). The distribution of individual cells along PC2 showed that most of the cells from the high permissive donor had a transcriptional state closer to a full activation state (Fig 1B, black dots). In contrast, individual cells from the low permissive donor showed higher heterogeneity along this axis (Fig 1B, red dots) with transcriptional states spreading between resting and 72h-activated bulk cell samples. These data are consistent with activation bringing the cells toward a permissive phenotype. Cells from the high permissive donor are more homogenous towards the permissive phenotype, while cells from the low permissive donor are more heterogeneous, with fewer cells that display the permissive phenotype. These data highlight the importance of single-cell approaches to investigate heterogeneity that are otherwise masked by bulk population analyses, as shown by the proximity of the two donor populations at 72h. We also used a supervised approach to evaluate the presence of different cell lineages by examining the expression of 63 genes broadly used to classify CD4^+^ T cell subpopulations (S5A Fig and S1 Table), including helper (Th1, Th2, Th17), regulatory (T-reg), and memory (Effector and Central memory) CD4^+^ T cells. Clustering of cells according to these markers did not show a clear patterning. Similar analysis was also performed with 1503 innate immunity genes [19] that led to no obvious clustering of cellular sub-populations (S5B Fig).

In summary, the unsupervised whole transcriptome PCA analysis and the supervised clustering using prototypical CD4^+^ T cell markers and innate immunity genes identified T cell activation as the major driver of cell heterogeneity and failed to identify the presence of distinct lineages of T cell subtypes in the samples. This opened the door to hypothesize that the cellular state, rather than cell fate and lineage, is the most significant contributor to permissiveness.

### Identification of cellular surface proteins as candidate markers for HIV permissiveness

**Fig 2:**
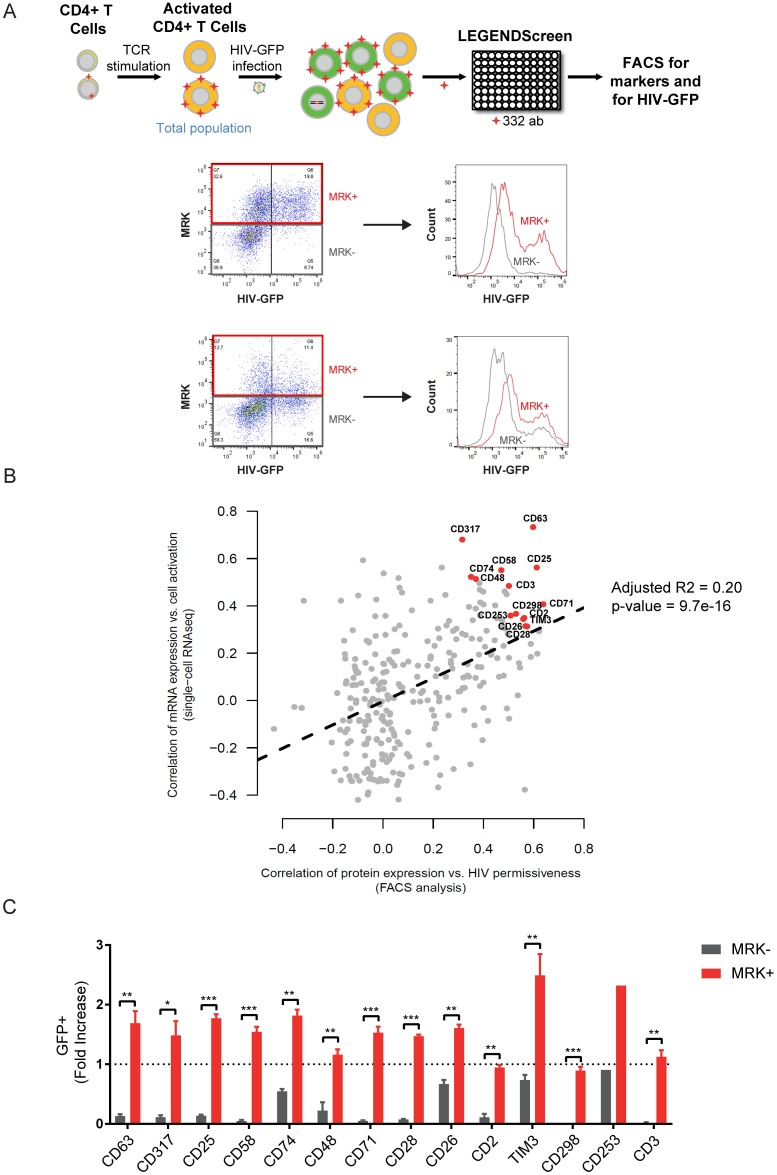
Screening for cellular markers of HIV permissiveness and CD4^+^ T cell activation. **(A)** Schematic representation of experimental procedure: CD4^+^ T cells were TCR-stimulated for 48h and transduced with HIV-GFP. After 24h, cells were stained with 332 PE-conjugated antibodies (LEGENDScreen) and analysed by FACS to evaluate the expression of each marker, as well as GFP expression (as a surrogate of successful viral infection). HIV permissiveness, i.e. GFP expression, was compared between cells expressing high levels of candidate marker (MRK^+^, red) and low levels of candidate marker (MRK^-^, grey). In the top example, the proportion of GFP^+^ cells was enriched in the MRK^+^ cell population; this candidate marker was thus positively correlated with HIV permissiveness. In contrast, the example of the bottom panel showed that the proportion of GFP^+^ cells was similar in both MRK^+^ and MRK^-^ cell populations, suggesting a poor correlation between marker expression and HIV permissiveness. **(B)** Correlation between single-cell transcriptome analysis and cell surface protein expression screen. From single-cell RNA-seq, a correlation was performed between the mRNA expression and cell activation for each candidate marker (y-axis). In parallel, the antibody screen allowed to correlate marker expression with HIV permissiveness (x-axis). Correlation between these two analyses (adjusted R-squared: 0.20; p-value = 9.7e-16) allowed to identify markers associated with HIV permissiveness and activation. Top candidate markers are highlighted in red. **(C)** Validation of top candidates as markers of HIV permissiveness. Activated CD4^+^ T cells were transduced with HIV-GFP and after 24h, GFP and marker expression was measured by FACS. Values correspond to proportion of GFP expressing cells and are normalized to one in the unsorted population (dash line; % of unsorted cell population between 8–20%). The proportion of GFP expressing cells was then assessed in both, MRK^+^ (red) and MRK^-^ (grey), gated subpopulations. Error bars indicate SEM and data shown are from 4 independent experiments with 4 different donors. ** corresponds to *P*<0.01; *** corresponds to *P*<0.001, from a paired t-test.

To identify protein biomarkers that would help sort and characterize the most permissive cells, we explored the hypothesis of T cell activation having a major role in HIV permissiveness. For this, we focused on genes coding for cell-surface proteins and whose RNA expression levels across single cells correlated with cell activation (PC2 axis, Fig 1B), and tested whether their protein levels at single-cell level associated with permissiveness to HIV (Fig 2). FACS analysis was used to investigate at single-cell resolution the expression level of surface proteins in activated CD4^+^ T cells and assessed how they associated with successful HIV infection as determined by GFP. This was accomplished with a large-scale surface molecule antibodies screening (Fig 2A, upper panel). Forty-eight hours after activation, CD4^+^ T cells from the original high permissive donor (donor #42) were infected with HIV-GFP and 24h later, a panel of 332 PE-conjugated antibodies (LEGENDScreen Human Cell Screening, BioLegend) was used to investigate by FACS the co-occurrence of cell surface proteins and successful infection (Fig 2A).

We assessed the Spearman rank correlation between each surface protein expression and GFP levels in each individual cell (S2 Table). These correlation values reflect the association of the marker with the cell permissiveness to productive HIV infection. We then determined the level of association of these genes to activation using single-cell RNA-seq data (PC2 in Fig 1B and S2 Table). Fig 2B shows that the candidate markers with the highest association with HIV permissiveness are also the ones with the highest association with activation state. Moreover, these results show that the main source of transcriptional heterogeneity across single-cells (as captured by the activation state in the second principal component from Fig 1B) drives HIV susceptibility as measured by the correlation of protein marker levels with GFP (adjusted R-squared: 0.20; p-value = 9.7e-16).

From this correlation analysis we selected 14 candidate markers: CD63, CD317, CD25, CD58, CD74, CD48, CD71, CD28, CD26, CD2, TIM3, CD298, CD253 and CD3; whose expression highly correlated with both T cell activation and HIV permissiveness (correlation ≥ 0.3 in both analyses and correlation ≥ 0.5 at least in one of the analyses, Fig 2B). The list includes, as expected, CD25 (IL-2 receptor), a canonical activation marker [28] associated with susceptibility to HIV [29–31]. The correlation between marker expression and HIV permissiveness was further validated in CD4^+^ T cells from four independent blood donors (Fig 2C). With the exception of CD253, expression of each candidate marker was correlated with permissive status of cells.

### CD4^+^ T cell sorting by selected markers prior to infection confirm their role as biomarkers for HIV permissiveness

To determine if the selected candidate proteins were predictive markers for HIV permissiveness, it was important to exclude that the association observed between the candidate markers and HIV permissiveness was not due to HIV infection *per se*. In fact, viral infection modulates expression of many cellular genes [2]. Moreover, marker expression (i.e. Mean Fluorescence Intensity (MFI) assessed by FACS) is often shifted or increased in the GFP^+^ population as compared to the GFP^-^ population (S6 Fig).

**Fig 3:**
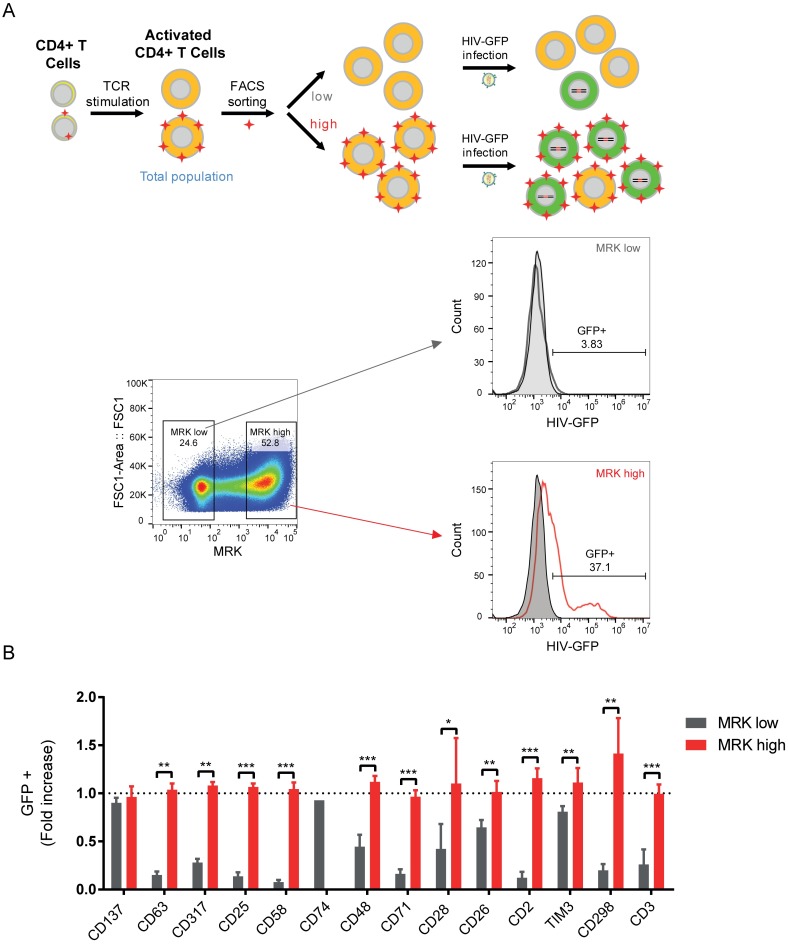
HIV permissiveness of cell populations expressing high and low levels of the selected markers. **(A)** Schematic representation of experimental procedure: CD4^+^ T cells were TCR-stimulated for 48h and FACS-sorted for each single marker in two populations, expressing high or low levels of the markers. These populations were then transduced with HIV-GFP and 48h post-transduction, GFP expression was evaluated by FACS. **(B)** HIV permissiveness in FACS sorted populations. Values correspond to GFP (%) fold increase in the high marker population (in red) and in the low marker population (in grey), as compared to unsorted population (dash line; % of unsorted cell population between 30–50%). Error bars indicate SEM and data shown is from 4 independent experiments with 4 different donors. ** corresponds to *P*<0.01, *** corresponds to *P*<0.001, from a paired t-test.

Therefore, activated CD4^+^ T cells were first sorted according to the expression of each candidate biomarker and then infected with HIV-GFP (Fig 3A). Sorting was performed based on MFI expression (high vs low) rather than the proportion of cells expressing or not the marker (+ *versus* -) (S7 Fig), as some of the selected markers were always present in CD4^+^ T cells while their MFI could change over time (CD48, CD2, CD298 and CD3; S8 Fig). As depicted in Fig 3B, marker-high populations were always more permissive than marker-low populations for all tested candidate biomarkers, except for CD74 which performed poorly for sorting and which was thus discarded from further experiments. Sorting based on CD137 protein expression was used as control and data showed a similar permissiveness to HIV infection between CD137^high^ and CD137^low^ populations.

### Identified biomarkers expression increase permissiveness in CD25 high-expressing cells

**Fig 4:**
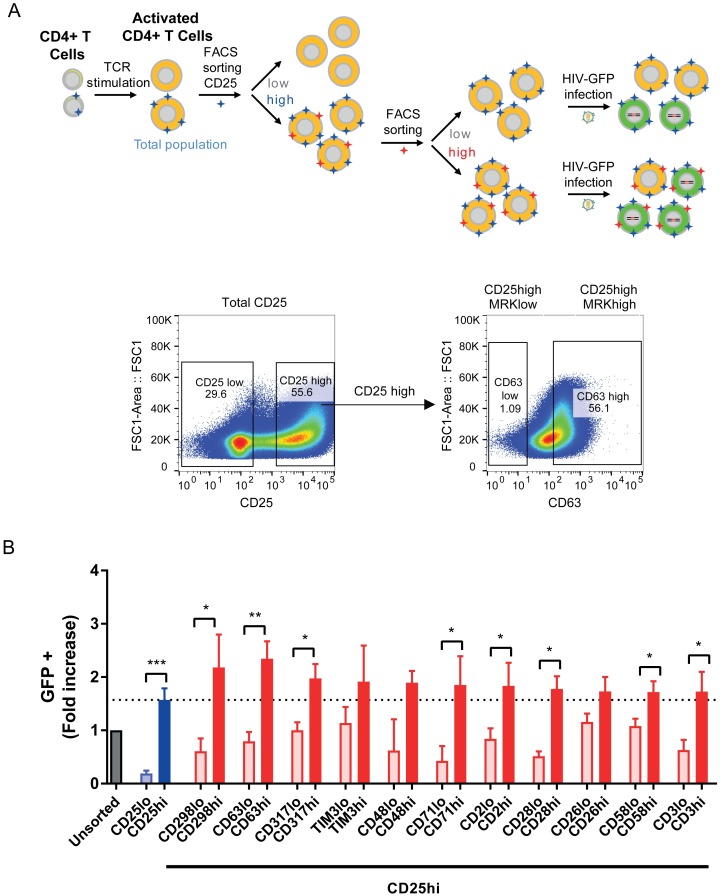
HIV permissiveness of CD25^high^CD4^+^ T cells expressing an additional candidate biomarker. **(A)** Schematic representation of experimental procedure: CD4^+^ T cells were TCR-stimulated for 48h and FACS sorted first for CD25 (high and low) and then the CD25^high^ population was further sorted for the expression of each of the other candidate markers (high expression *versus* low expression). These populations were then transduced with HIV-GFP and 48h post-transduction, GFP expression was evaluated by FACS. Gating for sorting of high and low expressing populations was always defined from the initial unsorted cell population. **(B)** HIV permissiveness in FACS sorted populations. The graph shows the permissiveness of the sorted population (fold increase from unsorted population). Sorted populations were CD25^low^ (light blue), CD25^high^ (dark blue), CD25^high^MRK^low^ (light red) and CD25^high^MRK^high^ (dark red). Values correspond to GFP (%) fold increase as compared to unsorted population (grey bar; unsorted population between 13–32% of GFP positive cells). Error bars indicate SEM and data shown is from 4 independent experiments with 4 different donors. * corresponds to *P*<0.05, ** corresponds to *P*<0.01, *** corresponds to *P*<0.001, from a paired t-test.

As CD25 is a strong activation marker, it might explain the whole phenotype of permissiveness [31]. In order to investigate the possible involvement of additional biomarkers, we first assessed how CD25 co-expressed with the other 11 biomarkers (S9 Fig) and whether these markers were able to further enrich for permissive cells in CD4^+^ T cells positive for CD25. To this end, TCR-stimulated CD4^+^ T cells were first sorted for CD25^high^ regardless of the presence or the absence of other markers (MRK), yielding a purified CD25^high^ CD4^+^ T cell population. This population was then further sorted for each of the other 11 markers to obtain cells CD25^high^MRK^low^ and CD25^high^MRK^high^. The different populations were then infected with HIV-GFP and analyzed by FACS, 48h post-infection (Fig 4A).

As expected, sorting by CD25^high^ enriched for cells permissive to HIV as compared to CD25^low^ and unsorted populations (2.4 fold and 1.6 fold, respectively). The CD25^high^ population can be further separated according to high and low expression level of additional markers, displaying further increased and decreased permissiveness, respectively. Indeed, use of additional markers in sorting resulted in CD25^high^MRK^high^ populations that increased the permissiveness to HIV as compared to CD25^high^MRK^low^ (ranging from 4.3 fold to 1.5 fold), confirming that all 11 markers contribute in addition to CD25 to further enrich for cells permissive to HIV (Fig 4B). Further evaluation showed that the increase in permissiveness to HIV observed in CD25^high^MRK^high^ populations as compared to CD25^high^ was also statistically significant for 8 out of the 11 candidates (S3 Table and S10 Fig).

Three markers, CD298, CD63 and CD317, with 2.2, 2.3 and 2.0 fold increase of permissive cells compared to unsorted cells, and 1.4, 1.5 and 1.3 fold compared to CD25^high^ cells, respectively, were selected for further analyses. Expression of these markers in different CD4^+^ T cells subsets, *i*.*e*. naïve *versus* memory cells 48h post-TCR stimulation, showed similar profiles, suggesting that the association of these markers with HIV permissiveness remains valid across cell lineages (S11 Fig).

### Combination of activation markers leads to an increase of HIV permissive cells

**Fig 5:**
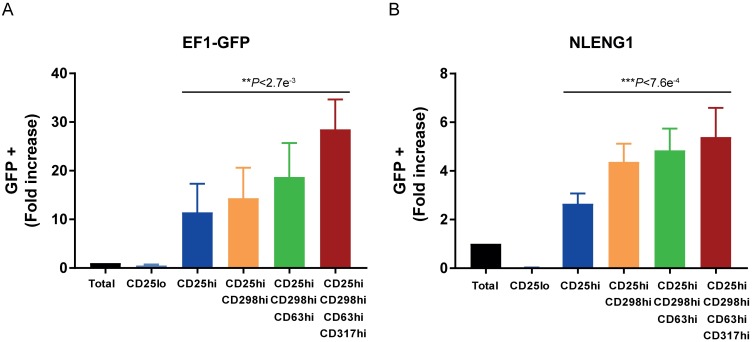
HIV permissiveness of CD4^+^ T cells highly expressing the top candidate biomarkers. CD4^+^ T cells were TCR-stimulated for 48h and FACS sorted sequentially for CD25^high^, CD298^high^, CD63^high^ and CD317^high^. The sorted populations were then transduced with HIV-GFP (EF1-GFP) **(A)** or the replication competent HIV virus expressing GFP (NLENG1) **(B)** Values correspond to GFP (%) fold increase as compared to unsorted population (unsorted population between 1–5% of GFP positive cells for HIV-GFP and 5–30% of positive cells for NLENG1). Error bars indicate SEM and data shown is from 4 independent experiments with 4 different donors in A and 5 independent experiments with 5 different donors in B. Statistical significance of the effect on the fold-increase of permissive cells compared to unsorted cells as a function of the number of additional activation markers used on top of CD25^high^ was evaluated in S4 Table using a linear regression model and led to significant p-values in both experiments (p-value<5e-03).

We tested the additive contribution of the 4 biomarkers, CD25, CD298, CD63 and CD317, to identify highly permissive cells. While the 4 markers generally co-express two by two (S12A Fig), only ~20% of activated CD4^+^ T cells highly co-express the 4 markers simultaneously (S12B Fig). These results led us to proceed with the sorting of the CD25^high^CD298^high^CD63^high^CD317^high^ population, where TCR-activated CD4^+^ T cells were sorted successively for high expression of CD25, CD298, CD63 and CD317 cells and then infected with HIV-GFP (EF1-GFP). As shown in Fig 5A, the progressive addition of markers increased in a statistically significant manner the amount of permissive cells with an increase of 28 fold from the unsorted population to the four marker combination (S4 Table and S13 Fig). The role of these markers to HIV permissiveness was confirmed with a full-length, replication-competent CXCR4-tropic virus expressing GFP (NLENG1), entering the cells via CXCR4-mediated entry rather than VSV-G (Fig 5B). Activated CD4^+^ T cells, from different donors, infected with NLENG1 showed a statistically significant increase of permissiveness to HIV infection up to 5.4 fold with the four markers (Fig 5B, S4 Table and S13 Fig). Taken together, these results indicate that the initial selected markers, CD25, CD298, CD63 and CD317, whose expression was associated with activation at single-cell level both at mRNA and protein levels, identify highly permissive cells. These cells are characterized by an intracellular environment supportive of HIV infection, from entry to protein expression. Due to technical limitations and increasing rarity of specific cell subpopulations, our analyses concentrated on 4 selected markers. However, the use of additional candidate markers should further improve the selection of the highest permissive cell (S14 Fig).

### Transcriptional features of HIV permissive cells

**Fig 6:**
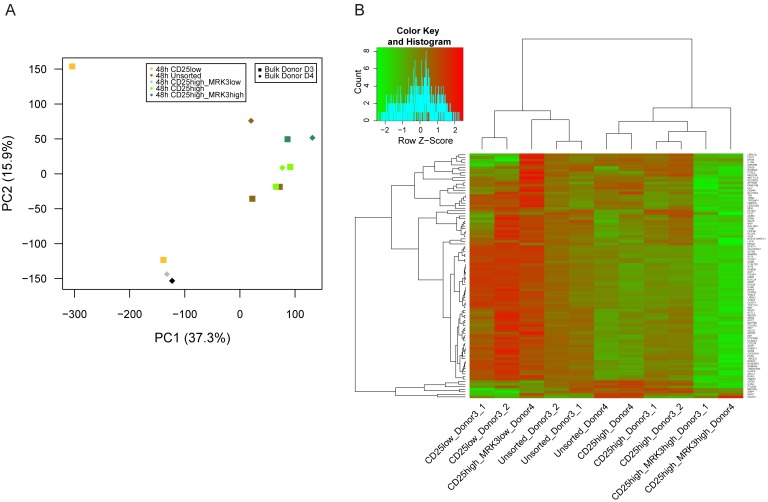
Transcriptome analysis of sorted subpopulations of bulk CD4^+^ T cells. **(A)** Principal Component Analysis of cell subpopulations of bulk CD4^+^ T cells at 48h after activation FACS sorted for: CD25^low^, CD25^high^MRK3^low^ (CD25^high^CD298^low^CD63^low^CD317^low^), CD25^high^ and CD25^high^MRK3^high^ (CD25^high^CD298^high^CD63^high^CD317^high^). The unsorted reference bulks are also included. PC1 placed samples according to their permissiveness to HIV infection from left (low permissive) to right (high permissive). **(B)** Heatmap clustering of the 96 genes differentially expressed among sorted CD25^high^ and CD25^high^MRK3^high^ (fold change higher or lower than 2 and adjusted p-value of < 0.001; DESeq2 test [32]). Complete hierarchical clustering of genes and cell samples was based on Pearson correlation. Color scale indicated in the legend corresponds to z-scores of gene expression levels expressed as the log10 of the number of library size-normalized reads per kilobase of exonic sequence, ranging from green (low) to red (high) expression. Downregulated genes are enriched in the type I interferon pathway (GO:0060337) and the defense response to virus (GO.0051607; S5 Table), and include important effectors such as: IFIT2, IFIT3, IRF7, ISG15, ISG20, MX1, RSAD2, XAF1, IFI44L and IL23A.

The availability of novel markers allowed the sorting of a unique cell subpopulation for characterization of the transcriptome. RNA was extracted from the sorted subpopulations according to MRK expression and used for RNA-Seq. Principal Component Analysis showed separation and ordering of cell subpopulations according to their permissiveness to HIV infection, i.e. from less to more permissive: CD25^low^, CD25^high^MRK3^low^ (CD25^high^CD298^low^CD63^low^CD317^low^), CD25^high^ and CD25^high^MRK3^high^ (CD25^high^CD298^high^CD63^high^CD317^high^) (Fig 6A).

Differential expression analyses among sorted CD25^high^ and CD25^high^MRK3^high^ subpopulations identified 96 genes (fold change higher or lower than 2 and adjusted p-value of < 0.001; DESeq2 test [32]), that could be involved in the observed differences in HIV permissiveness. 95 genes were differentially downregulated in CD25^high^MRK3^high^ as compared to CD25^high^ (Fig 6B and S5 Table). Functional enrichment analysis established that downregulated genes were in the type I interferon pathway (GO:0060337, False Discovery Rate 4.17e-06) and the defense response to virus (GO.0051607, FDR = 1.32e-04) (S6 Table). Genes involved included important effectors such as: IFIT2, IFIT3, IRF7, ISG15, ISG20, MX1, RSAD2, XAF1, IFI44L, and IL23A. In sharp contrast, the 95 genes appeared as highly expressed in both CD25^low^ and CD25^high^MRK3^low^ subpopulations. These observations further stress the relationship between activation state and innate immunity defense, in line with recent work [1]. Although not among the 96 genes differentially expressed between sorted CD25^high^ and CD25^high^MRK3^high^ populations (fold change higher or lower than 2 and adjusted p-value of < 0.001), the expression profile of the 5 prototypical HIV restriction factors still clustered the subpopulations similarly, i.e. according to the permissiveness phenotype (S15 Fig).

## Discussion

CD4^+^ T cells are the main target of HIV infection and the major cellular reservoirs of HIV *in vivo*. Understanding the heterogeneity of these cells in terms of permissiveness to HIV is crucial for the characterization of HIV infection and pathogenesis. In this study, we exploited the natural heterogeneity within and across individuals to explore differences between permissive and non-permissive cells by single-cell RNA-seq. This approach allowed the characterization of response to TCR activation as the main driver of heterogeneity. Moreover, coupling single-cell RNA-seq analysis with a high-throughput antibody screen for cell surface protein expression led to the identification of biomarkers for the HIV permissive cell.

The analysis of single-cell RNA-seq allowed assessing the dynamic nature of TCR activation. We observed a continuum of individual cells transiting intermediary activation states that recapitulated transcriptional changes occurring from resting CD4^+^ T cells to late times of activation. We then identified sets of genes reflecting such heterogeneity. Supervised analyses of RNA-seq levels of cell surface markers prototypical of CD4^+^ T cell subpopulations as well as of innate immunity genes did not make apparent the existence of distinct lineages of cells. Taken together, our results indicate that the activation state was the main component of CD4^+^ T cell transcriptional heterogeneity.

Activation state of CD4^+^ T cells has been extensively linked to HIV permissiveness in population, bulk samples. It is well known that quiescent cells are refractory to HIV infection [5, 33]. Here, we have further associated activation and permissiveness at single cell resolution characterizing both phenotypes at transcriptional and proteomic levels. In addition, the use of a high-throughput antibody screen assessing expression of more than 300 cell surface proteins allowed investigating protein expression with HIV infection success. This combined analysis showed that the more a gene and the encoded protein correlate with activation, the more they correlate with permissiveness.

The top candidate markers that correlated with both activation and permissiveness were further validated as predictors for HIV permissiveness. Expression of cell surface proteins allowed for sorting live cells from activated CD4^+^ T-cell populations from different donors. Individual markers identified cell subpopulations with greater susceptibility to HIV infection than the general activated cell population. The experimental approach examined the role of CD25 expression for HIV permissiveness and identified additional surface markers, which combined with CD25 led to the identification of a unique population of CD4^+^ T cells that is highly permissive to HIV. In addition to CD25, the best predictive biomarkers for permissiveness were CD298, CD63 and CD317.

CD63 is a tetraspanin associated with the membranes of intracellular vesicles. CD63 can also be expressed at cell surface, possibly upon T cell activation, thereby promoting sustained T cell activation and cell proliferation [34]. CD63 has been described to participate in HIV infection in early and late steps of the HIV life cycle, both in macrophages and CD4^+^ T cells [35–38]. CD317, also known as BST-2 or tetherin, is a lipid raft associated protein that restricts HIV infection by retaining nascent virions at the cell surface and preventing their release [39]. CD298 is the beta3 subunit of the Na,K ATPase, known to maintain the electrochemical gradients of sodium and potassium across the plasma membrane [40]. CD298 has been associated as regulator of T cell activation independently of the ATPase alpha subunit [41]. Interestingly, CD298 was shown to be an antagonist of BST-2 by binding and inducing BST-2 degradation and therefore facilitating HIV replication [42].

The transcriptional profile of CD25^high^CD298^high^CD63^high^CD317^high^ expressing cells revealed a specific signature as compared to the CD25^high^ cell population, characterized by the down-regulation of 96 genes, most of which are involved in innate immunity. These results are consistent with previous model suggesting that activated CD4^+^ T cells are more permissive to HIV infection than resting CD4^+^ T cells in part due to reduced innate immune responses [1]. The specific signature of this cell population highlights the potential of single-cell RNA-seq analysis as a general pipeline for biomarker identification.

In conclusion, single-cell RNA-seq allowed the investigation of CD4^+^ T-cell heterogeneity prior to infection and led to the identification of markers for permissiveness to HIV cellular infection. Their combined use serves to identify a cellular state that defines the “HIV permissive cell”. A similar approach should be used to identify the biomarkers of the resting cell predicting its activation potential, and thus its permissive phenotype. Finally, the relevance of these highly permissive cells *in vivo*, *i*.*e*. *w*ill these cells more likely be infected and then become latent and contribute to the reservoir or will they more likely die and be depleted, remains to be elucidated.

## Materials and methods

### Ethics statement

All blood donors have provided written informed consent. All samples were anonymized.

### Cell samples, isolation and culture conditions

Peripheral Blood Mononuclear Cells (PBMCs) from different healthy blood donors were purified by Ficoll gradient separation, using Leucosep tubes (Greiner), followed by CD4^+^ T cell isolation using negative selection and magnetic separation, according to manufacturer’s instructions (Stemcell Technologies). Total CD4^+^ T cells were isolated using EasySep Human CD4^+^ T Cell Enrichment Kit, naïve cells were isolated using EasySep Human Naïve CD4^+^ T Cell Enrichment Kit and memory CD4^+^ T cells were isolated using Human Memory CD4^+^ T Cell Enrichment Kit. Cell enrichment yielded a purity of CD4^+^T cell populations of up to 96%.

Non-activated CD4^+^ T cells were maintained in RPMI-1640 culture medium (Gibco) supplemented with 10% heat-inactivated fetal bovine serum (FBS). T-cell receptor (TCR)-mediated activation of CD4^+^ T cells was performed using CD3/CD28 co-stimulation in presence of IL-2 (100 U/ml, R&D Systems). For this, anti-CD3 antibodies (4 μg) were plated in 1 ml PBS per well of a 6-well plate and incubated overnight at 4°C. Wells were washed once with PBS and cells were added in each well at 2x10^6^ cells/ml in OpTmizer CTS T-Cell Expansion serum-free medium (Gibco), supplemented with 1 μg/ml anti-CD28 antibodies (BD Biosciences).

### Virus production and infection

For the HIV-based lentiviral vector EF1-GFP/VSV-G (named HIV-GFP) production, 2.5 million of HEK293T cells (from [43]) per 10 cm dishes were transfected with 15 μg of pWPI-EF1α-GFP [44], 10 μg of psPAX2 packaging plasmid (gift from Didier Trono (Addgene plasmid # 12260)) and 5 μg pMD2G coding for the VSV-G envelope [45], using calcium phosphate transfection kit (Life Technologies). HIV-GFP particles were then harvested 48h after transfection, filtered through 0.45 μm filters and concentrated by filtration on Centricon units (Centricon Plus-70/100K, Millipore). Replication-competent CXCR4-tropic NLENG1 viruses were produced by transfecting HEK293T cells with pNLENG1-IRES (gift from David N. Levy, [46]) using jetPEI (Polyplus transfection), following manufacturer’s instructions and collected 48h later. Of note, NLENG1 derives from pNL4-3 and contains *gfp* as a fluorescent reporter gene (eGFP-IRES-nef). Viral titers were measured by HIV-1 p24 Enzyme-linked immunosorbent assay (ELISA) kit (Innogenetics).

Primary CD4^+^ T cells (100,000 cells) were infected by spinoculation (1500 x g, 25°C, 1h30) in the presence of 4 μg/ml polybrene with 300 ng of HIV-GFP or 25 ng NLENG1. After spinoculation cells were washed and maintained for 48h or 72h in culture with OpTmizer CTS T-Cell Expansion serum-free medium in the presence of 100U/ml of IL-2.

### Antibody screen (LEGENDScreen Human Cell Screening)

CD4^+^ T cells from donor #42 (from a previous study [3]) were TCR-activated for 48h as described above and infected with HIV-GFP. After 24h of infection, CD4^+^ T cells were incubated with the LEGENDScreen Human Cell Screening PE-conjugated Antibodies (BioLegend), according to manufacturer’s recommendations. Briefly, 100,000 cells were stained with each provided PE-antibody for 30 min at 4°C, according to the manufacturer’s protocol, washed with PBS-BSA (1%), fixed with 200 μl of CellFIX (BD Biosciences) and analyzed by FACS Gallios (Beckman Coulter). Marker expression and GFP reporter expression data were analyzed using FlowJo software (Tree Star).

### Flow cytometry analysis of surface markers and cell sorting

CD4^+^ T cells were collected at 0h, 24h, 48h, and 72h after TCR activation and analyzed by FACS for longitudinal expression assays. Co-expression analysis of multiple biomarkers was performed at 48h post-activation only. Briefly 100,000 cells were used for each staining, washed twice with PBS-BSA, and incubated at 4°C for 30 min with the different surface antibodies (S7 Table). Cells were then washed twice with PBS-BSA, fixed with CellFIX and analyzed by flow cytometry using FACS BD Accuri C6 (BD Biosciences). Data was analyzed using FlowJo software.

Staining for cell sorting was performed on a cell suspension of 10–40 million CD4^+^ T cells, in 250–500 μl of RoboSep buffer (Stemcell Technologies). After cell washing, cells were incubated with the different antibodies (S8 and S9 Tables) at 4°C for 30 min, washed and resuspended in Robosep Buffer (1–2 ml) prior to sorting on MoFlo Astrios (Beckman Coulter Life Sciences; Flow Sorting Facility, University of Lausanne).

### Single-cell RNA-seq

Cells from the two donors (#42 and #123, from a previous study [3]) were TCR-activated as previously described, and maintained in 2 ml OpTmizer expansion medium with 5% FBS and100 U/ml IL-2 for 72h. Dead cells were removed by centrifugation on a 3 ml Percoll gradient at 800 x g for 10 minutes. Cells were washed, counted and resuspended in PBS. Cells were either used for bulk RNA-Seq or loaded to the Fluidigm C1 platform for single-cell capture and single-cell RNA-seq library generation according to the manufacturer’s protocol. Briefly, a cell suspension of approximately 300,000 cells/ml was introduced into the medium size chip (10–17 μm plate), suitable to capture cells of 10–17 ± 2 μm, and thus able to capture activated cells (usually ranging from 10 to 13 μm). After cell separation and capture, empty or debris-occupied wells were identified by microscope visualisation and discarded from subsequent analysis. cDNA libraries were then produced directly and automatically on the chip with Clontech SMARTer Ultra Low RNA kit for Illumina using manufacturer-provided protocols. Illumina libraries were constructed in 96-well plates using the Illumina Nextera XT DNA Sample Preparation kit according to a protocol supplied by Fluidigm and sequenced on HiSeq2500 machine (Illumina), with 50 bp paired-end, 14 libraries multiplexed per lane.

### Population RNA-seq

RNA from bulk samples was extracted using Illustra RNAspin kit (GE Healthcare). mRNA-Seq library preparation was performed with TruSeq RNA sample prep kit (Illumina) starting with capture of polyA-containing transcripts, followed by cluster generation (TruSeq single-end cluster generation kit, Illumina) and high-throughput sequencing on Illumina HiSeq2500 (Genomics Technology Facility, University of Lausanne). 100 cycles single-end sequencing was performed for all bulk samples except for the high permissive/low permissive donors that used 50 cycles paired-end sequencing. Additional bulk samples from resting and activated CD4^+^ T cells used in the PCA analysis were from published work [47].

### Bioinformatics analysis

Sequenced reads obtained were cleaned before alignment using cutadapt v0.9.5 [48] to remove the adapter if present at the 3' end of the read with an overlap between the read and the adapter equal or higher than 13 bases, and prinseq v0.20 to remove: i) low quality nucleotides at the 3′ or 5′ end of the reads (PHRED score<6); ii) reads with mean PHRED score lower than 20; iii) poly-A/T tail with a minimum length of 13 either at the 5’-end or the 3’-end; iv) poly-N tail with a minimum length of 2 either at the 5'-end or at the 3’-end. Cleaned reads were aligned to the human reference genome with STAR aligner v2.3.0 [49] using the Ensembl gene GRCh37 release 70 annotation file. RUM aligner [50] was used for the first 8 bulk libraries indicated in S10 Table. The number of reads per gene was quantified with HTSeq-count v.0.6.1 [51], with parameters mode = union and type = exon. An average library size of 59,862,736 and 6,618,513 uniquely mapped reads in the bulk and single-cell libraries was obtained respectively. For downstream analysis, log-transformation of gene expression values was performed as the log10 of the number of library size-normalized reads per kilobase of exonic sequence. A pseudo-count of 1 was added previous to the log10 transformation to avoid NA’s: log10(RPKM*59862736/1000000+1). The index of bulk and single-cell RNA-seq libraries, the per-gene raw read-counts matrix and the described log-transformed matrix are provided in S10, S11 and S12 Tables. One of the single-cell libraries from the high resistant donor (“poolT12_2”) appeared as an outlier in initial exploratory analyses and was discarded from downstream analyses. Analysis of transcriptional heterogeneity across single cells was performed using Sincell Bioconductor package [27]. Differential expression analyses used DESeq2 bioconductor package [32]. Functional enrichment analysis was performed using STRING with default parameters [52]. An R script with the code necessary to reproduce all bioinformatics results and figures is provided as S1 File. Versions of R-packages used are detailed in at the end of S1 File.

### Statistical analysis

Statistical analyses were performed using the Prism software (v6.0aGraphPad). All comparisons of HIV permissiveness in non-overlapping subpopulations (single populations or single and multiple sorting populations) were performed using paired t-test.

To assess the significance of the use of additional markers to CD25, thus comparing nested subpopulations, we fitted for each selected candidate marker a linear regression model on the fold-increase observed when adding to the CD25^high^ subpopulation (taken as a basal reference level) the second marker high, while accounting for the Donor effects in the regression model. To make the model robust to departures from normality of the residuals distribution, we used 3 alternative transformations of the dependent variable: absolute rank, relative rank within donor and log-transformation of the fold increase.

## Supporting information

S1 Fig Selection of cell donors for single-cell RNA-seq. The selection of a high and a low permissive donor was based on permissiveness to HIV-GFP and growth index (cell count on day 14/cell count on day 7 post-activation). Two donors were selected, identified here as “42” and “123”, with differences in level of HIV permissiveness (~40% and ~10% GFP^+^ infected cells, respectively) while showing similar growth capacity.
(PDF)

S2 Fig Distribution of pairwise similarities among the whole transcriptome of the individual cells from a donor. The figure shows the distribution of Spearman correlation values between the log-transformed gene expression levels of all pairwise comparisons among the individual cells from the same individual. Gene expression levels are the log10 of the number of library size-normalized reads per kilobase of exonic sequence (Methods). Distributions have medians of 0.72 and 0.59 and standard deviations of 0.09 and 0.12 for the high and low permissive donor, respectively (Wilcoxon-rank sum two-sided test p-value < 2.2e-16).
(PDF)

S3 Fig Hierarchies of the transcriptomes of individual cells. Cell-state hierarchies of the high permissive **(A)** and low permissive **(B)** donors assessed using Sincell Bioconductor package. Hierarchies are based on the first two dimensions of a PCA performed on the log-transformed gene expression values (Methods) followed by Iterative Mutual Clustering (parameter k = 4) as described in [27]. Assessment of hierarchies was restricted to the 3558 genes significantly variable across individual cells, assessed on the library size normalized read count matrix and performing Winsorization as described in http://pklab.med.harvard.edu/scw2014/subpop_tutorial.html. **(C)** and **(D)**: Statistical support for the cell-state hierarchies represented in A and B, respectively. Figures represent the distribution of similarities (Spearman rank correlations; median 0.92 and 0.96 for c and d, respectively) between the reference cell-state hierarchy and the 100 hierarchies obtained when 100 random sets of 50% of genes are subsampled, as described in Juliá *et al*. 2015 [27].
(PDF)

S4 Fig Equivalence across the Principal Component Analysis (PCA) axes of single-cell RNA-seq libraries. **(A)** Principal component analysis of the log-transformed gene expression levels of 85 and 81 single-cell RNA-seq libraries from the high permissive (black) and low permissive (red) donors. Gene expression levels are the log10 of the number of library size-normalized reads per kilobase of exonic sequence (Methods). **(B)** Correlation between the PC2 cell coordinates from Fig 1B and the PC1 cell coordinates from S4 Fig **Panel A** (Pearson correlation = 0.9994).
(PDF)

S5 Fig Clustering and heatmap of the expression levels across individual cells from high (left) and low (right) permissive donors does not cluster single cells according to cell subpopulation or innate immunity gene expression. **(A)** 63 genes (S1 Table) commonly used to classify CD4^+^ T cell subpopulations, including helper (Th1, Th2, Th17), regulatory (T-reg), and memory (Effector and Central memory) CD4^+^ T cells, and **(B)** 1503 innate immunity genes as described in previous work [19]. Single cell samples are displayed by columns as labeled on the bottom, and genes are displayed by rows. Complete hierarchical clustering of genes and cell samples was based on Spearman correlation of the log10 of the number of library size-normalized reads per kilobase of exonic sequence (Methods). Color scale indicated in the legend corresponds to such log-transformed expression levels, ranging from green (low) to red (high) expression.
(PDF)

S6 Fig Impact of HIV-GFP infection on marker expression. FACS plots of candidate marker expression in CD4^+^ T cells after 48h TCR-activation followed by 24h mock (grey) or HIV-GFP (green) infection. This figure is representative of 3 independent experiments.
(PDF)

S7 Fig Gating strategy for FACS sorting. **(A)** Gates defining high and low expressing markers were set on the total population. When the population was clearly separated in two subpopulations, the gates were placed according to the medium-high population density corresponding to the red-yellow areas (example, top panel with CD25). When the population was homogenous, the gates were placed to the extreme expression levels, corresponding to low population density and thus outside the red-yellow area (example, lower panel with CD317). **(B)** The gates set on the total population were used for successive sorting with the 4 top candidate markers.
(PDF)

S8 Fig Expression of selected markers in CD4^+^ T cells over time upon TCR activation. The expression of the selected markers was followed by surface staining and FACS analysis before and 1, 2, 3 and 4 days after TCR activation. Each graph indicates the percentage of marker expression (black) and the Mean Fluorescence intensity (MFI, orange). The last graph shows the viability of cells from three independent donors and their mean (black line). Error bars indicate SEM and data shown is from 3 independent experiments with 3 different donors.
(PDF)

S9 Fig Co-expression of selected markers with CD25. Columns left panels: FACS plots showing the co-expression of selected markers with CD25 in CD4^+^ T cells after 48h post-activation. Columns right panels: Pearson correlation of the corresponding FACS plot (marker *versus* CD25).
(PDF)

S10 Fig Enrichment of permissiveness in sorted cell subpopulations. Fold-increase of permissive cells compared to unsorted cells after use of a second marker (CD25^high^Marker^high^) to the CD25^high^ populations for the 4 donors evaluated in Fig 4B. The increase in permissiveness to HIV observed in CD25^high^MRK^high^ populations as compared to CD25^high^ was evaluated in S3 Table.
(PDF)

S11 Fig Enrichment in HIV permissiveness is not dependent on the memory or naïve lineage. Naïve and memory CD4^+^ T cell populations were purified from PBMCs by negative selection, and cell phenotype was confirmed by FACS analysis on CD45RA (naïve cells) or CD45RO (memory cells). Cells from the two different subsets were then activated for 48h and infected with HIV-GFP. After 24h, staining and FACS analysis were performed to evaluate the co-expression of GFP and the different markers in naïve (top panel) and memory cells (right panel). This figure is representative of 3 independent experiments.
(PDF)

S12 Fig Co-expression of the top selected markers. **(A)** FACS plots showing co-expression of selected markers, two by two in CD4^+^ T cells after 48h post-activation. (**B)** Co-expression of the 4 selected markers (CD25^high^, CD298^high^, CD63^high^, CD317^high^).
(PDF)

S13 Fig Enrichment of permissiveness in sorted cell subpopulations. Fold-increase of HIV permissive cells compared to unsorted cells after use of additional markers (Marker^high^) to the CD25^high^ populations for the donors evaluated in Fig 5A (left panel) and Fig 5B (right panel). The increase in permissiveness to HIV of CD25^high^ cells as a function of the number of additional MRK^high^ populations was statistically significant in both experiments (S4 Table).
(PDF)

S14 Fig Cell selection with additional candidate markers further improves the capture of the highest permissive cells. CD4^+^ T cells were TCR-stimulated for 48h and FACS sorted sequentially for CD25^high^, CD298^high^, CD63^high^, CD317^high^ and CD2^high^. The sorted populations were then transduced with HIV-GFP (EF1-GFP) **(A)** or CXCR-4 tropic NLENG1 **(B)**, and HIV permissiveness was assessed by FACS. Values correspond to GFP (%) fold increase as compared to unsorted population. Error bars indicate SEM and data shown is from 3 independent experiments with 3 different donors.
(PDF)

S15 Fig Heatmap clustering of the 5 prototypical HIV restriction factors across different sorted subpopulations. Complete hierarchical clustering of genes and cell samples was based on Pearson correlation. Color scale indicated in the legend corresponds to z-scores of gene expression levels expressed as the log10 of the number of library size-normalized reads per kilobase of exonic sequence, ranging from green (low) to red (high) expression.
(PDF)

S1 Table List of 63 prototypical genes characterizing CD4^+^ T cell subpopulations and used for the generation of heatmaps in S5A Fig. (TXT)

S2 Table Correlation values represented in Fig 2B. First column in the table corresponds to the Spearman rank correlation between surface protein expression and GFP levels in individual cells (see text). Second column corresponds to the correlation of the corresponding gene expression levels with PC2 in Fig 1B, as determined by single-cell RNA-seq.
(TXT)

S3 Table Statistical analysis (P-values) assessing the effect on the fold-increase of permissive cells compared to unsorted cells caused by the addition of a second marker (MRK^high^) to the CD25^high^ population, as determined by a linear regression model accounting for the donor effects (S10 Fig). To make the test robust to departures from normality of the residuals distribution, 3 alternative transformations of the dependent variable were used: absolute rank, relative rank within donor and log-transformation of the fold increase.
(XLSX)

S4 Table Statistical analysis (P-values) assessing the effect on the fold-increase of permissive cells compared to unsorted cells as a function of the number of additional activation markers used on top of CD25^high^ (*i*.*e*. 1, 2 or 3 markers), as determined by a linear regression model accounting for the donor effects (S13 Fig). To make the test robust to departures from normality of the residuals distribution, 3 alternative transformations of the dependent variable were used: absolute rank, relative rank within donor and log-transformation of the fold increase. The p-values were significant (p-value<5e-03) in both experiments under the 3 transformations evaluated.
(XLSX)

S5 Table 96 genes differentially expressed between sorted CD25^high^ and CD25^high^MRK3^high^ (CD25^high^CD298^high^CD63^high^CD317^high^) subpopulations (fold change higher or lower than 2 and adjusted p-value of < 0.001; DESeq2 test). (TXT)

S6 Table Functional enrichment results of the 96 differentially expressed genes reported in S5 Table. Enrichment analysis was performed using STRING [52] with default parameters and the whole human genome as a reference background.
(XLS)

S7 Table List of antibodies used for flow cytometry for screening validation of the candidate markers (Fig 2C). (XLSX)

S8 Table List of antibodies used for FACS sorting of single and CD25^high^MRK sortings candidate markers (Figs 3B and 4B). (XLSX)

S9 Table List of antibodies used for FACS sorting of the four best candidate markers (Fig 5). (XLSX)

S10 Table Index of bulk and single-cell RNA-seq libraries. (TXT)

S11 Table Raw read counts of the RNA-seq libraries included in this work, as described in S10 Table. (ZIP)

S12 Table Gene expression matrix expressed as the log10 of the number of library size-normalized reads per kilobase of exonic sequence (Methods): log10(RPKM*59862736/1000000+1). (ZIP)

S13 Table Gene lengths used in this work for the RPKM assessment. (TXT)

S14 Table Mapping between gene Ensembl identifiers, gene names and biotypes used in this work. (TXT)

S1 File R script with the necessary code to reproduce the main figures and tables from the bioinformatics analysis. Versions of the R-packages used are detailed at the end of S1 File.
(R) Click here for additional data file.
