# Supplementary figures and images for: Single-cell analysis identifies cellular markers of the HIV permissive cell
Source: PLoS Pathog. 2017 Oct 26;13(10):e1006678. doi: 10.1371/journal.ppat.1006678 (PMC5658171; doi:10.1371/journal.ppat.1006678)

S1 Fig

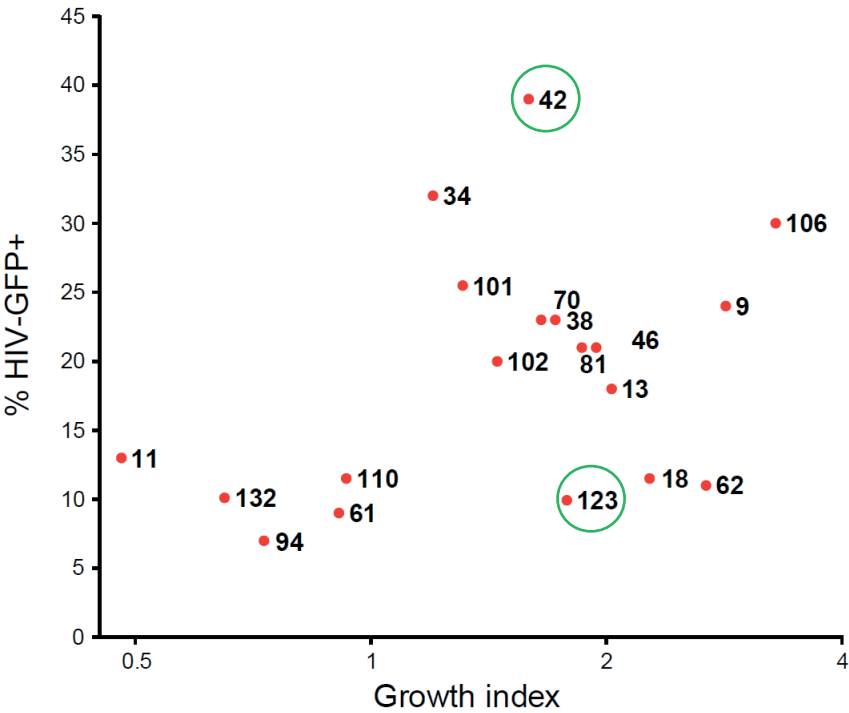

Supplement: S1 Fig — The selection of a high and a low permissive donor was based on permissiveness to HIV-GFP and growth index (cell count on day 14/cell count on day 7 post-activation). Two donors were selected, identified here as “42” and “123”, with differences in level of HIV permissiveness (~40% and ~10% GFP+ infected cells, respectively) while showing similar growth capacity. (PDF) [file ppat.1006678.s001.pdf]

S2 Fig

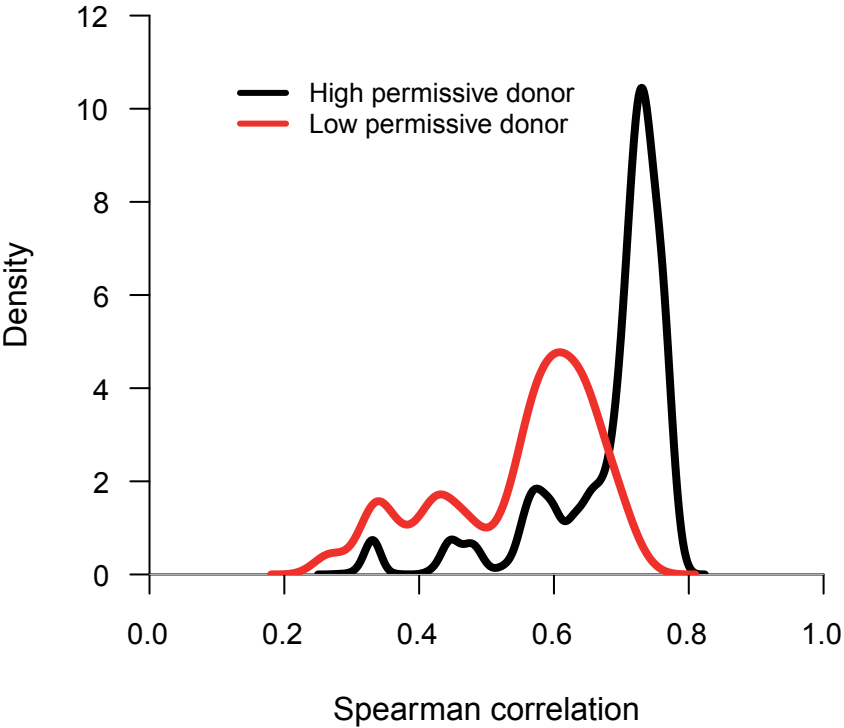

Supplement: S2 Fig — The figure shows the distribution of Spearman correlation values between the log-transformed gene expression levels of all pairwise comparisons among the individual cells from the same individual. Gene expression levels are the log10 of the number of library size-normalized reads per kilobase of exonic sequence (Methods). Distributions have medians of 0.72 and 0.59 and standard deviations of 0.09 and 0.12 for the high and low permissive donor, respectively (Wilcoxon-rank sum two-sided test p-value < 2.2e-16). (PDF) [file ppat.1006678.s002.pdf]

S3 Fig

A

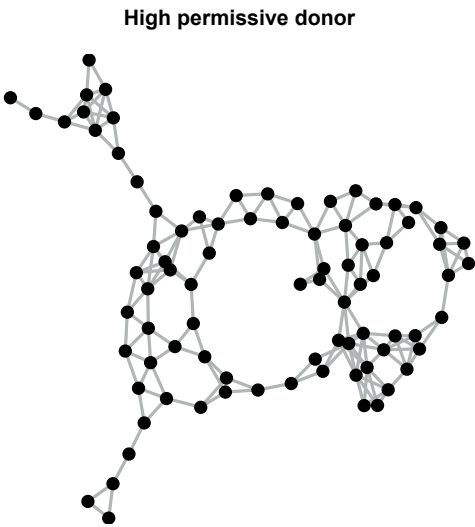

B

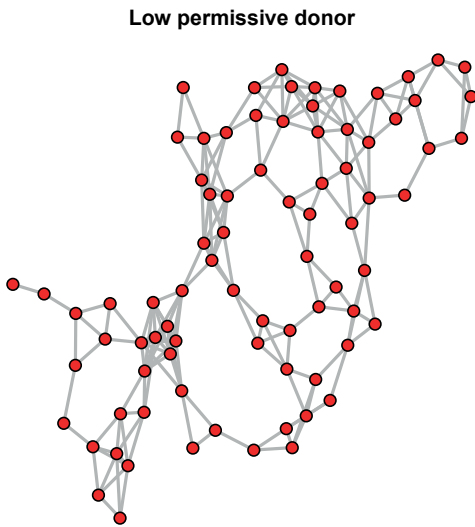

C

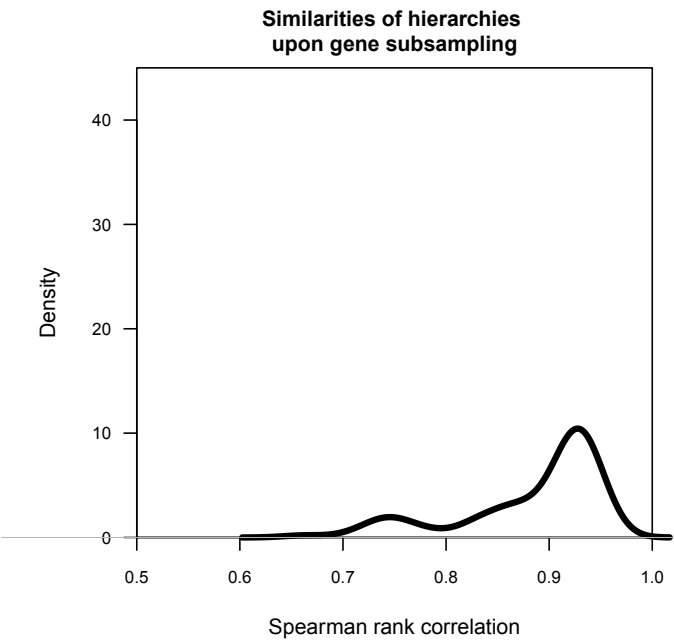

D

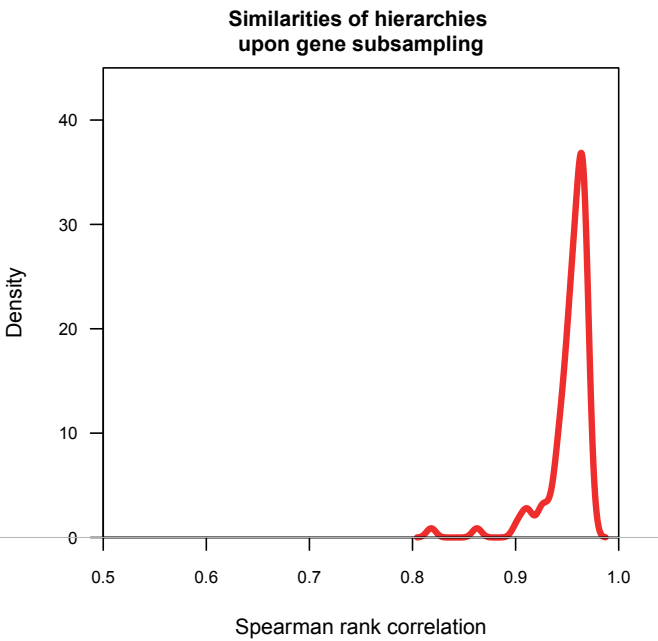

Supplement: S3 Fig — Cell-state hierarchies of the high permissive (A) and low permissive (B) donors assessed using Sincell Bioconductor package. Hierarchies are based on the first two dimensions of a PCA performed on the log-transformed gene expression values (Methods) followed by Iterative Mutual Clustering (parameter k = 4) as described in [27]. Assessment of hierarchies was restricted to the 3558 genes significantly variable across individual cells, assessed on the library size normalized read count matrix and performing Winsorization as described in http://pklab.med.harvard.edu/scw2014/subpop_tutorial.html. (C) and (D): Statistical support for the cell-state hierarchies represented in A and B, respectively. Figures represent the distribution of similarities (Spearman rank correlations; median 0.92 and 0.96 for c and d, respectively) between the reference cell-state hierarchy and the 100 hierarchies obtained when 100 random sets of 50% of genes are subsampled, as described in Juliá et al. 2015 [27]. (PDF) [file ppat.1006678.s003.pdf]

S4 Fig

A

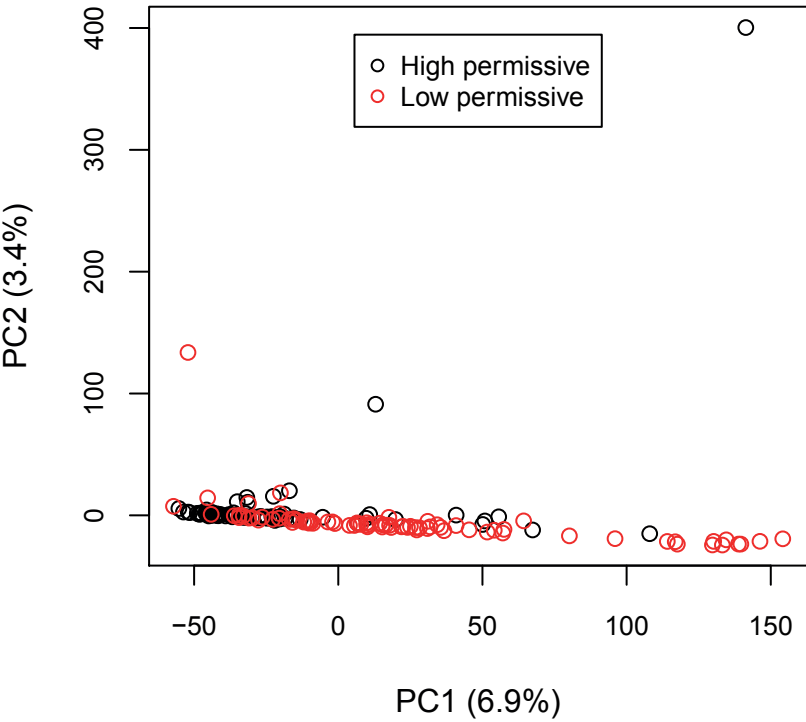

B

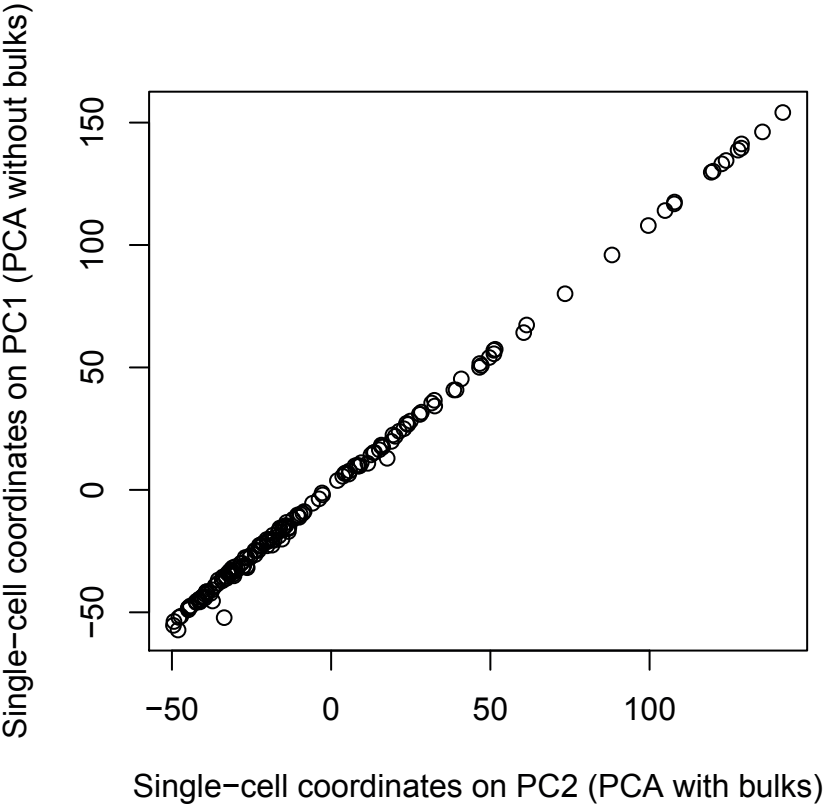

Supplement: S4 Fig — (A) Principal component analysis of the log-transformed gene expression levels of 85 and 81 single-cell RNA-seq libraries from the high permissive (black) and low permissive (red) donors. Gene expression levels are the log10 of the number of library size-normalized reads per kilobase of exonic sequence (Methods). (B) Correlation between the PC2 cell coordinates from Fig 1B and the PC1 cell coordinates from S4 Fig Panel A (Pearson correlation = 0.9994). (PDF) [file ppat.1006678.s004.pdf]

S5 Fig

A

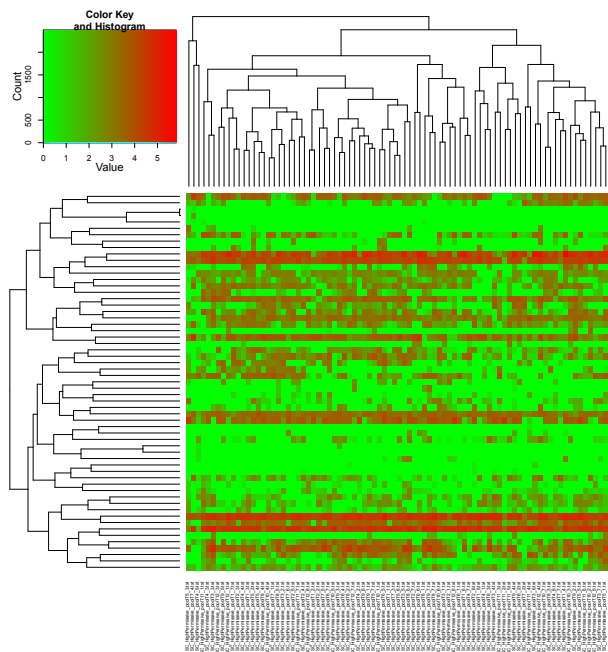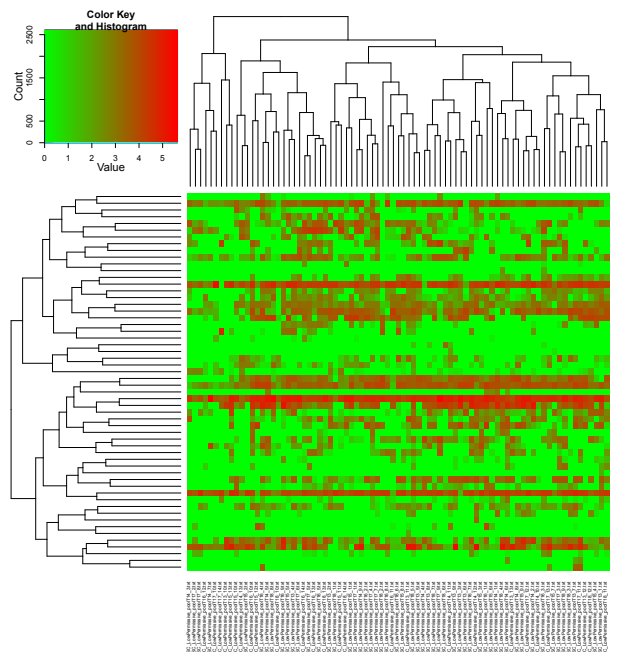

B

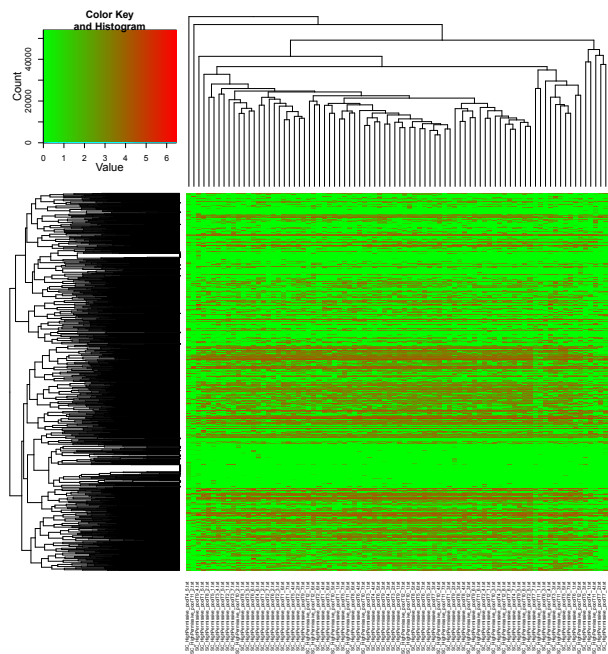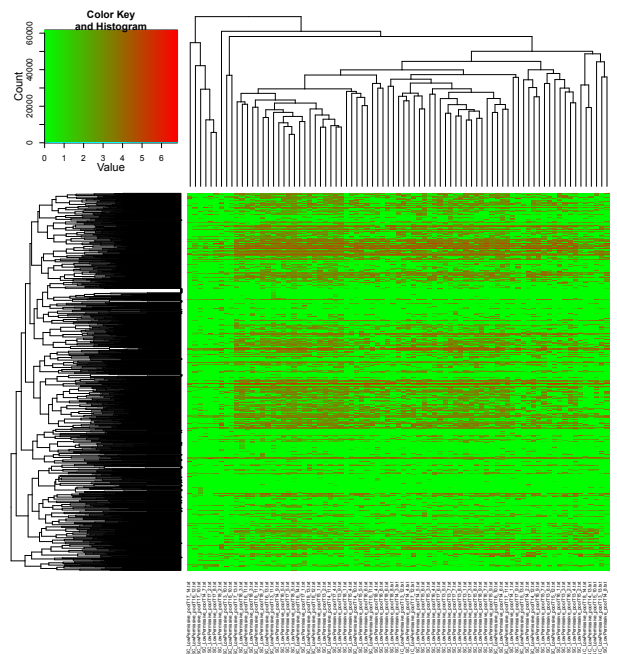

Supplement: S5 Fig — (A) 63 genes (S1 Table) commonly used to classify CD4+ T cell subpopulations, including helper (Th1, Th2, Th17), regulatory (T-reg), and memory (Effector and Central memory) CD4+ T cells, and (B) 1503 innate immunity genes as described in previous work [19]. Single cell samples are displayed by columns as labeled on the bottom, and genes are displayed by rows. Complete hierarchical clustering of genes and cell samples was based on Spearman correlation of the log10 of the number of library size-normalized reads per kilobase of exonic sequence (Methods). Color scale indicated in the legend corresponds to such log-transformed expression levels, ranging from green (low) to red (high) expression. (PDF) [file ppat.1006678.s005.pdf]

S6 Fig

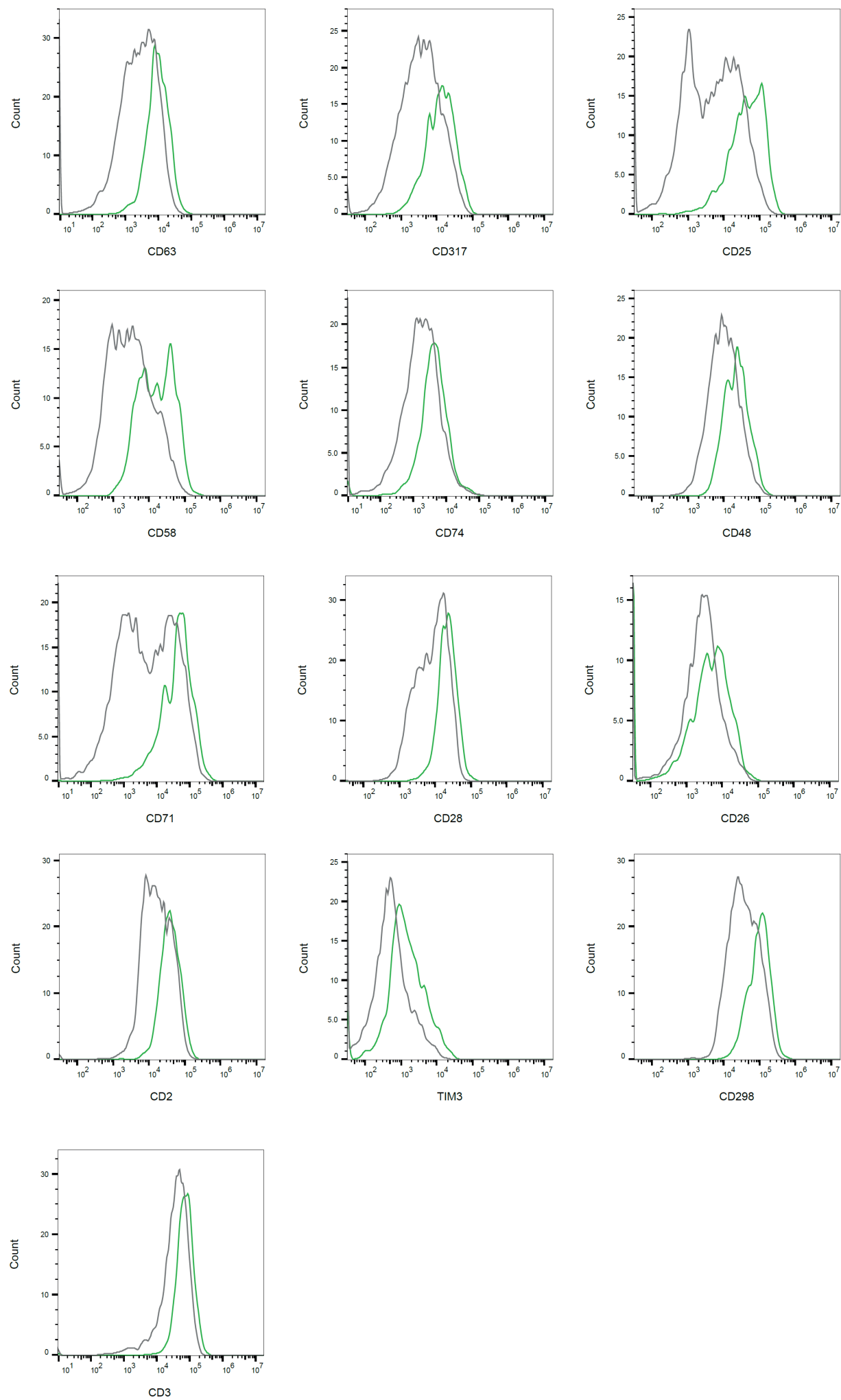

Supplement: S6 Fig — FACS plots of candidate marker expression in CD4+ T cells after 48h TCR-activation followed by 24h mock (grey) or HIV-GFP (green) infection. This figure is representative of 3 independent experiments. (PDF) [file ppat.1006678.s006.pdf]

A

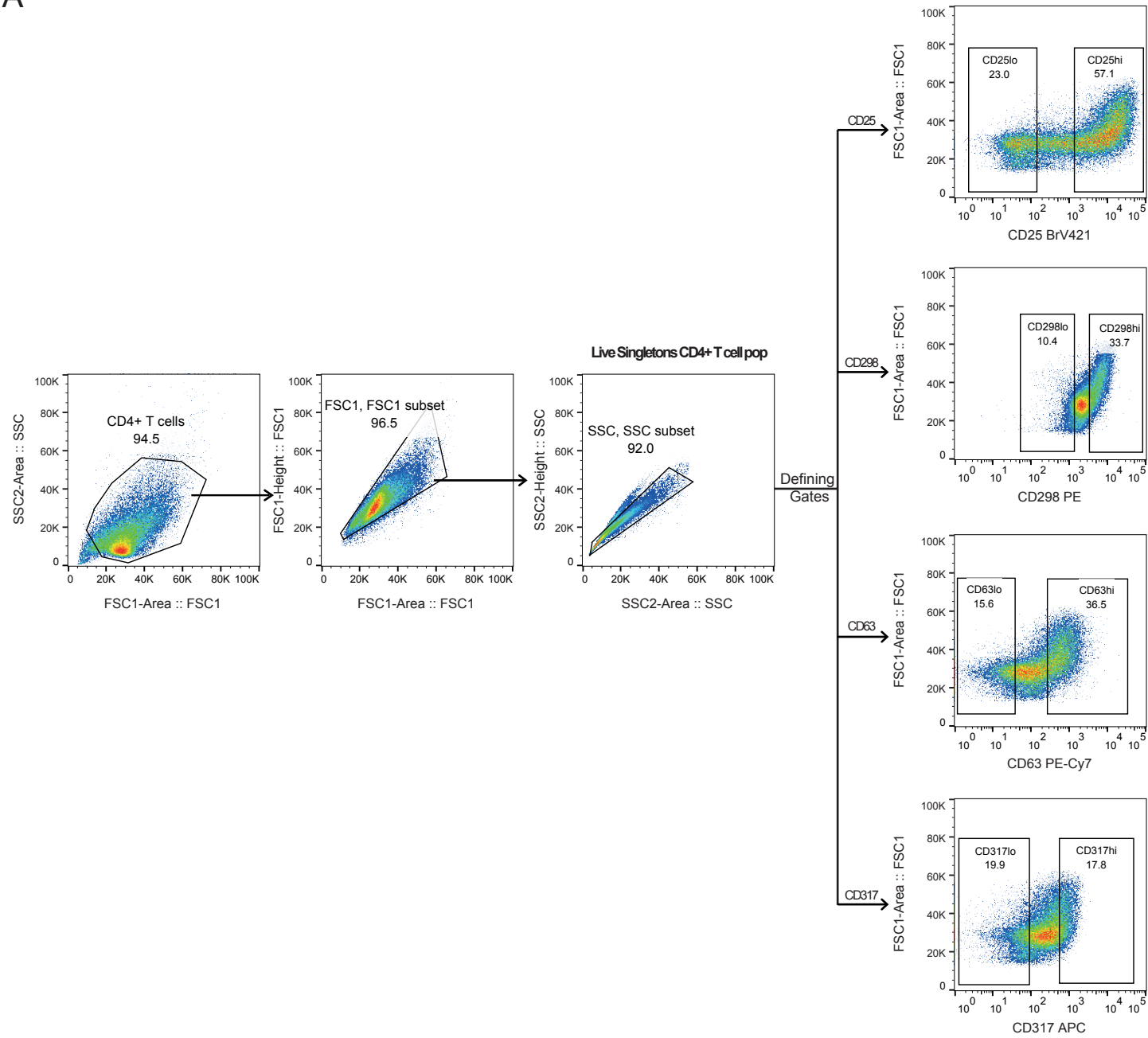

B

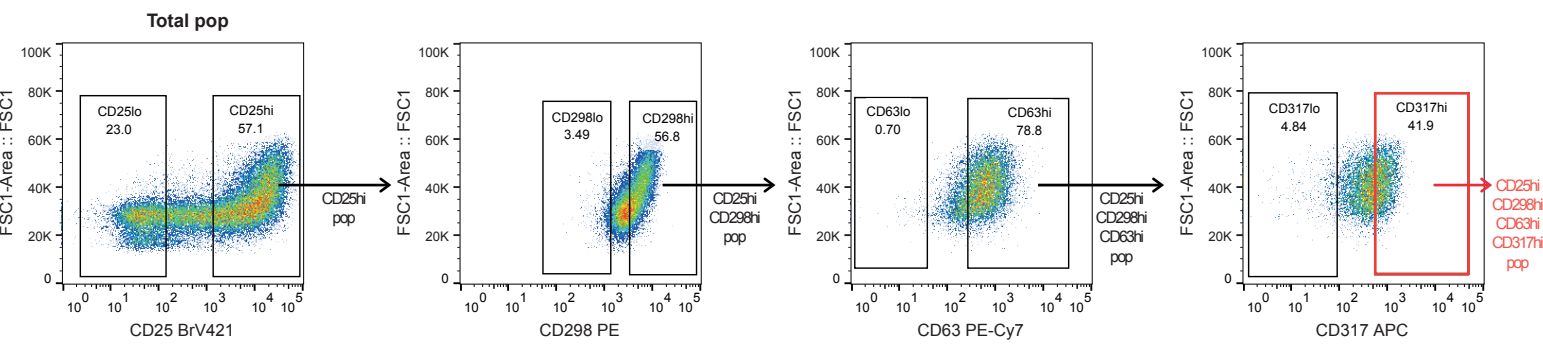

Supplement: S7 Fig — (A) Gates defining high and low expressing markers were set on the total population. When the population was clearly separated in two subpopulations, the gates were placed according to the medium-high population density corresponding to the red-yellow areas (example, top panel with CD25). When the population was homogenous, the gates were placed to the extreme expression levels, corresponding to low population density and thus outside the red-yellow area (example, lower panel with CD317). (B) The gates set on the total population were used for successive sorting with the 4 top candidate markers. (PDF) [file ppat.1006678.s007.pdf]

S8 Fig

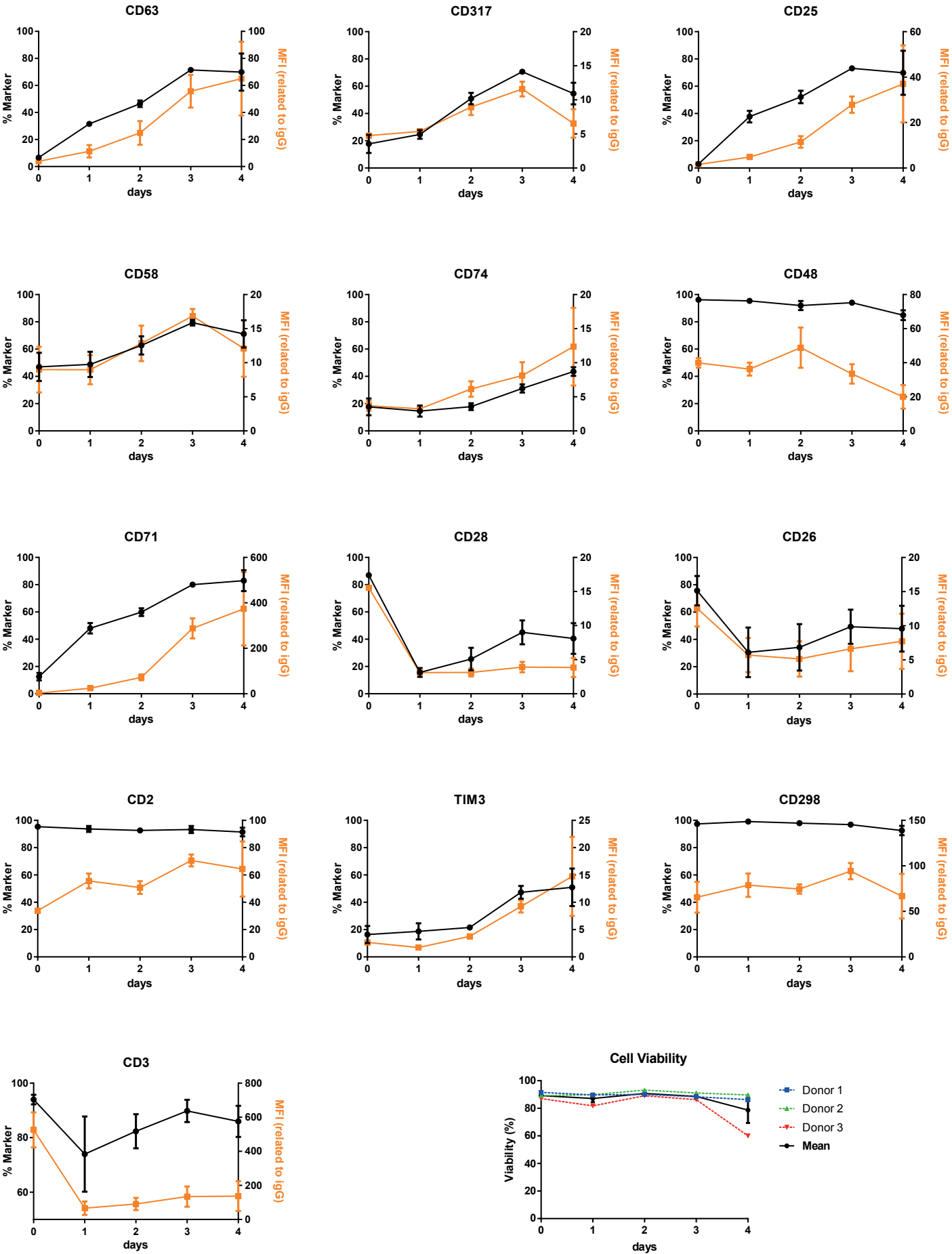

Supplement: S8 Fig — The expression of the selected markers was followed by surface staining and FACS analysis before and 1, 2, 3 and 4 days after TCR activation. Each graph indicates the percentage of marker expression (black) and the Mean Fluorescence intensity (MFI, orange). The last graph shows the viability of cells from three independent donors and their mean (black line). Error bars indicate SEM and data shown is from 3 independent experiments with 3 different donors. (PDF) [file ppat.1006678.s008.pdf]

S9 Fig

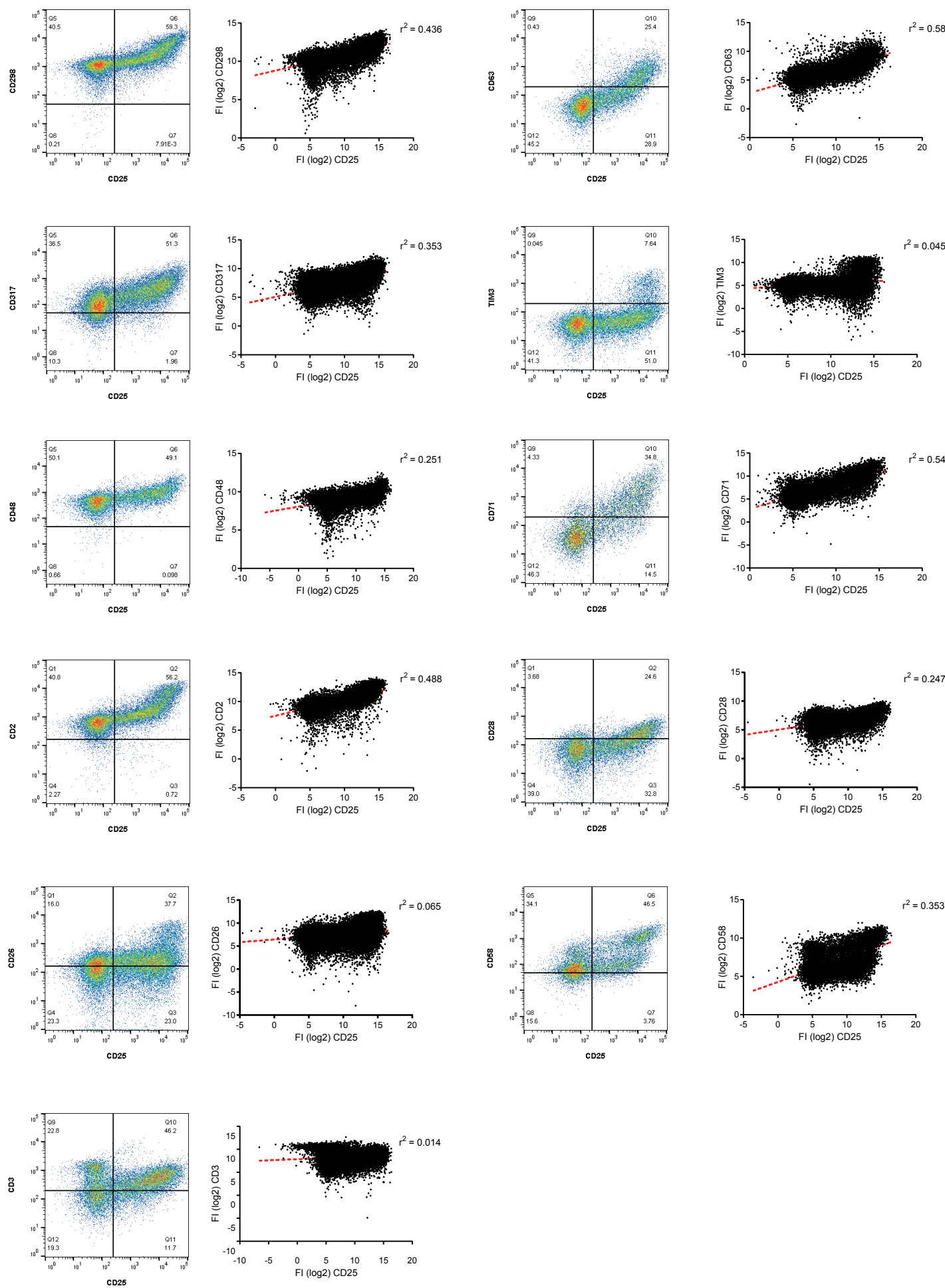

Supplement: S9 Fig — Columns left panels: FACS plots showing the co-expression of selected markers with CD25 in CD4+ T cells after 48h post-activation. Columns right panels: Pearson correlation of the corresponding FACS plot (marker versus CD25). (PDF) [file ppat.1006678.s009.pdf]

S10 Fig

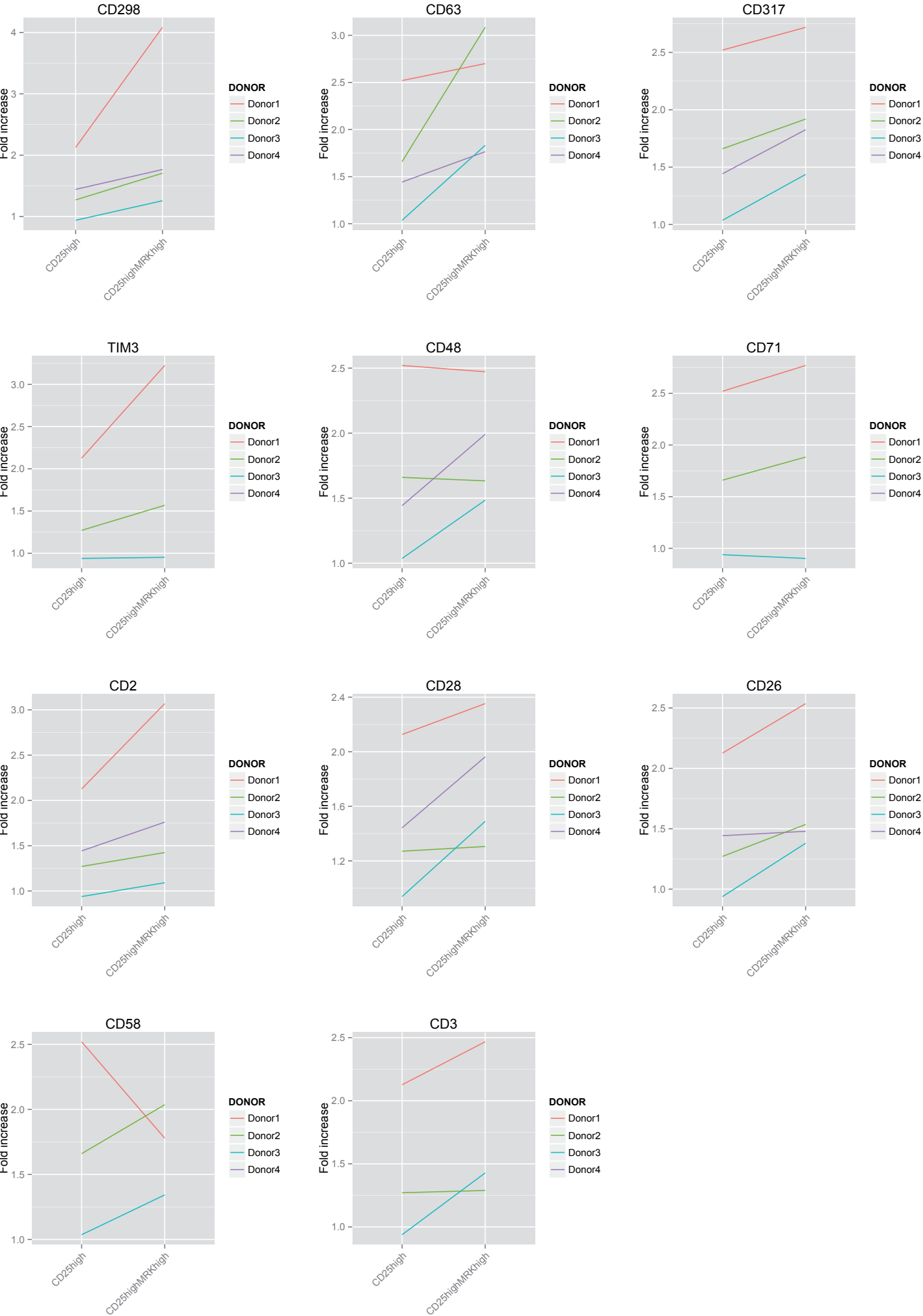

Supplement: S10 Fig — Fold-increase of permissive cells compared to unsorted cells after use of a second marker (CD25highMarkerhigh) to the CD25high populations for the 4 donors evaluated in Fig 4B. The increase in permissiveness to HIV observed in CD25highMRKhigh populations as compared to CD25high was evaluated in S3 Table. (PDF) [file ppat.1006678.s010.pdf]

S11 Fig

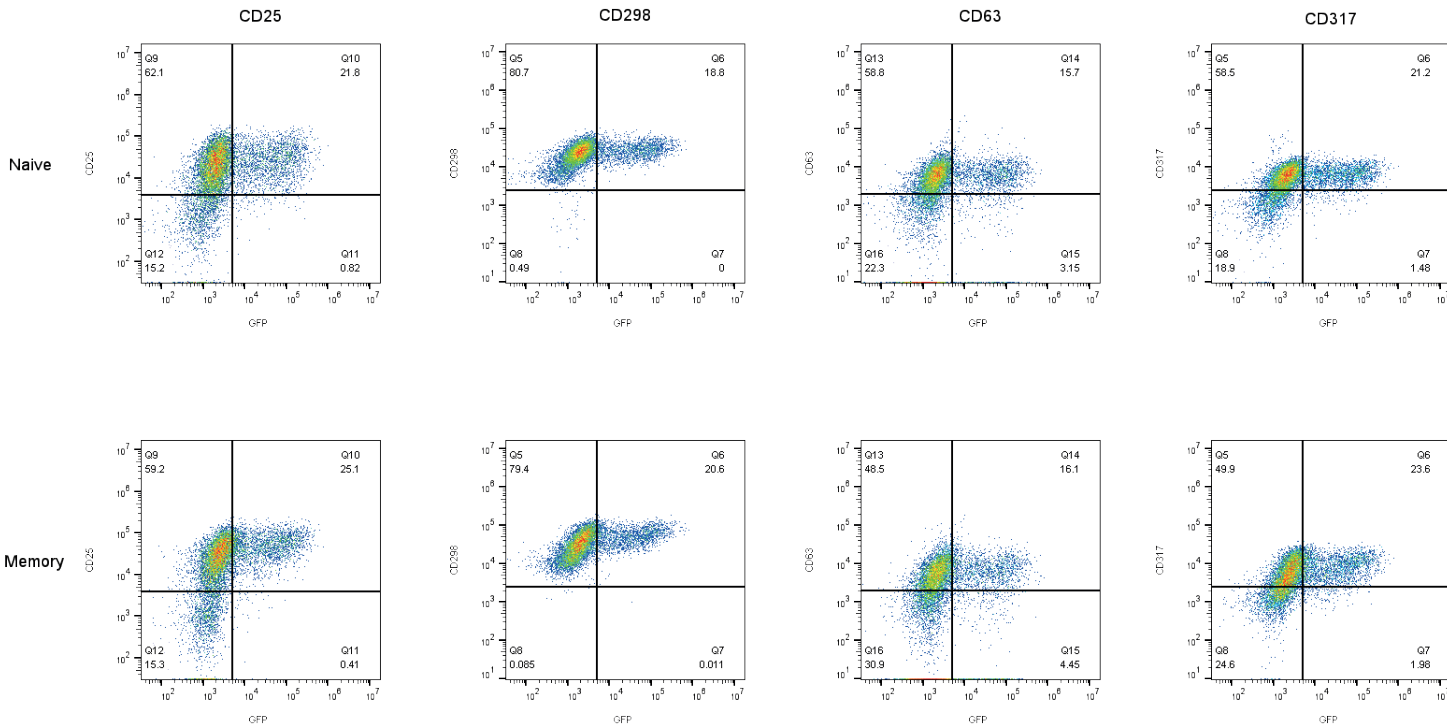

Supplement: S11 Fig — Naïve and memory CD4+ T cell populations were purified from PBMCs by negative selection, and cell phenotype was confirmed by FACS analysis on CD45RA (naïve cells) or CD45RO (memory cells). Cells from the two different subsets were then activated for 48h and infected with HIV-GFP. After 24h, staining and FACS analysis were performed to evaluate the co-expression of GFP and the different markers in naïve (top panel) and memory cells (right panel). This figure is representative of 3 independent experiments. (PDF) [file ppat.1006678.s011.pdf]

S12 Fig

A

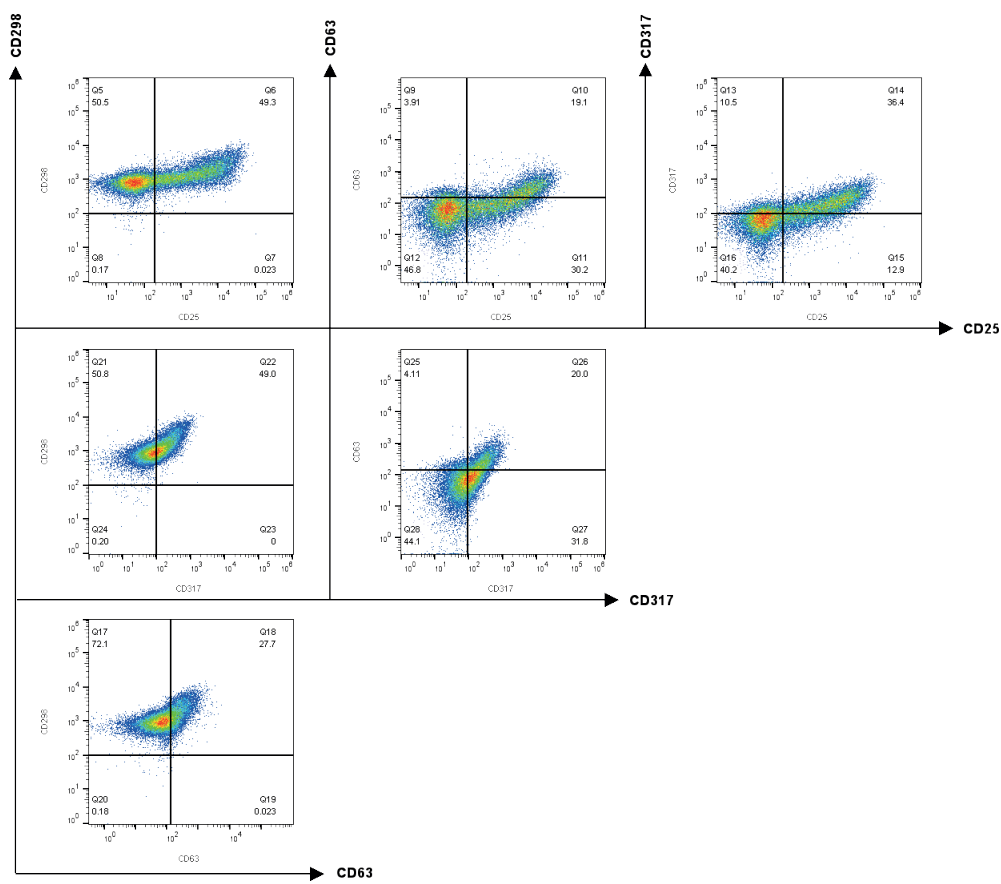

B

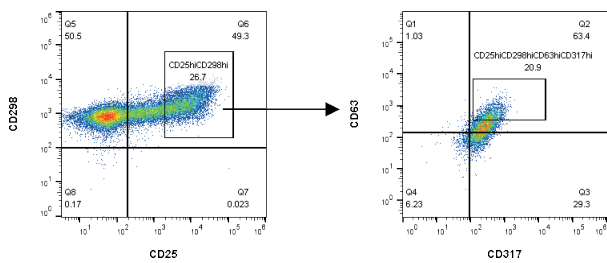

Supplement: S12 Fig — (A) FACS plots showing co-expression of selected markers, two by two in CD4+ T cells after 48h post-activation. (B) Co-expression of the 4 selected markers (CD25high, CD298high, CD63high, CD317high). (PDF) [file ppat.1006678.s012.pdf]

S13 Fig

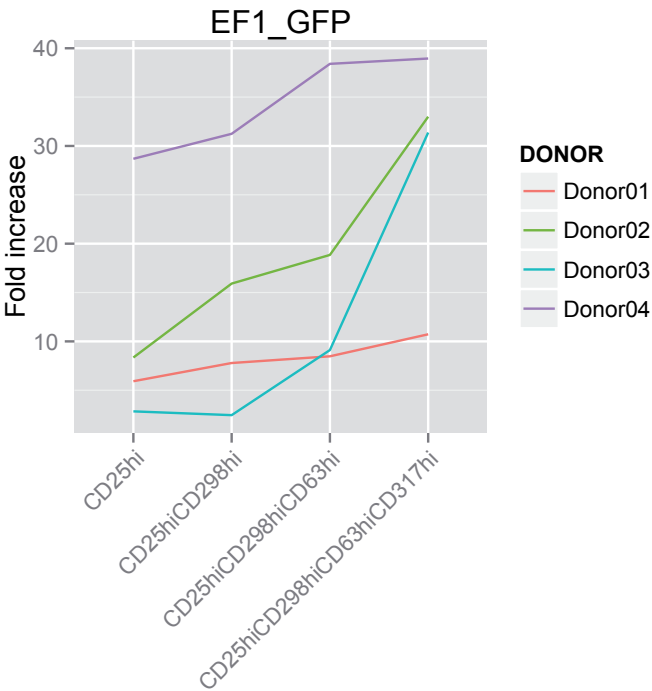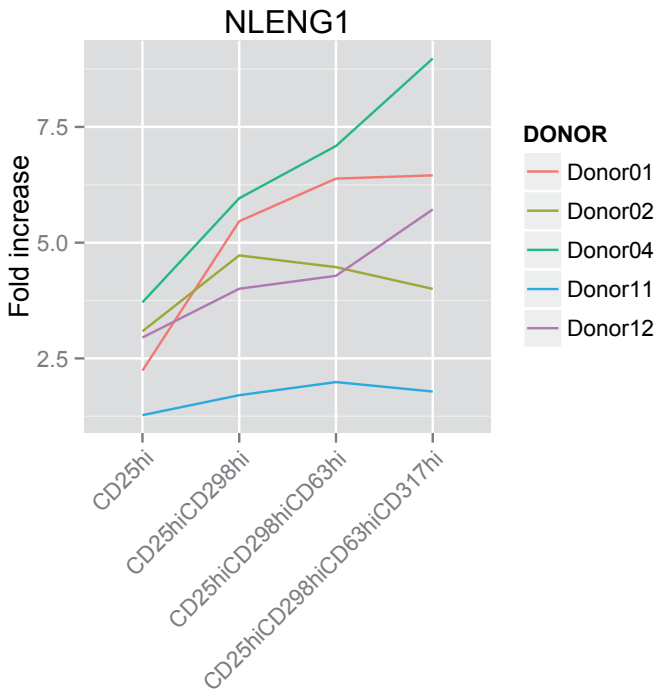

Supplement: S13 Fig — Fold-increase of HIV permissive cells compared to unsorted cells after use of additional markers (Markerhigh) to the CD25high populations for the donors evaluated in Fig 5A (left panel) and Fig 5B (right panel). The increase in permissiveness to HIV of CD25high cells as a function of the number of additional MRKhigh populations was statistically significant in both experiments (S4 Table). (PDF) [file ppat.1006678.s013.pdf]

S14 Fig

A

EF1-GFP

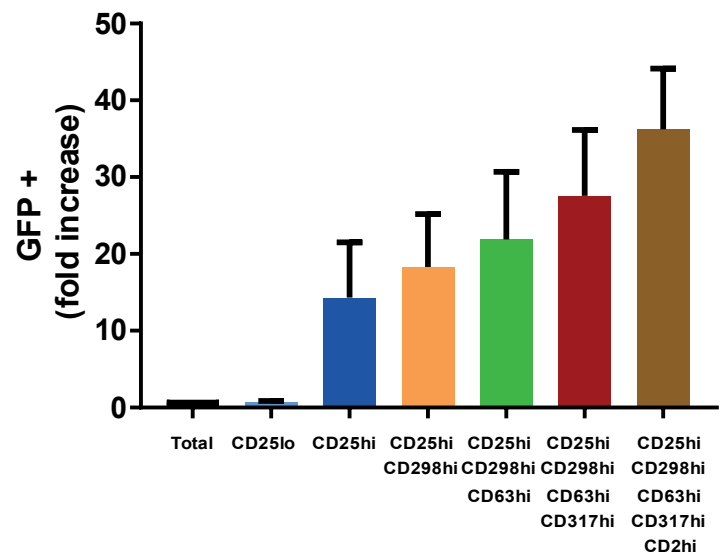

B

NLENG1

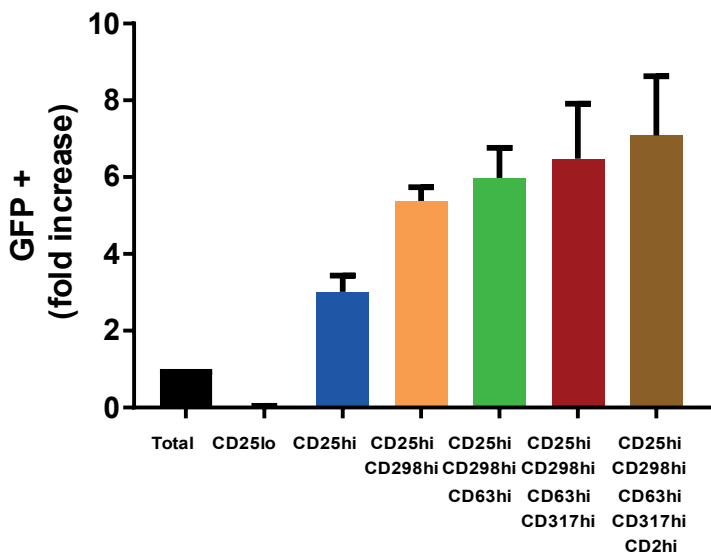

Supplement: S14 Fig — CD4+ T cells were TCR-stimulated for 48h and FACS sorted sequentially for CD25high, CD298high, CD63high, CD317high and CD2high. The sorted populations were then transduced with HIV-GFP (EF1-GFP) (A) or CXCR-4 tropic NLENG1 (B), and HIV permissiveness was assessed by FACS. Values correspond to GFP (%) fold increase as compared to unsorted population. Error bars indicate SEM and data shown is from 3 independent experiments with 3 different donors. (PDF) [file ppat.1006678.s014.pdf]

S15 Fig

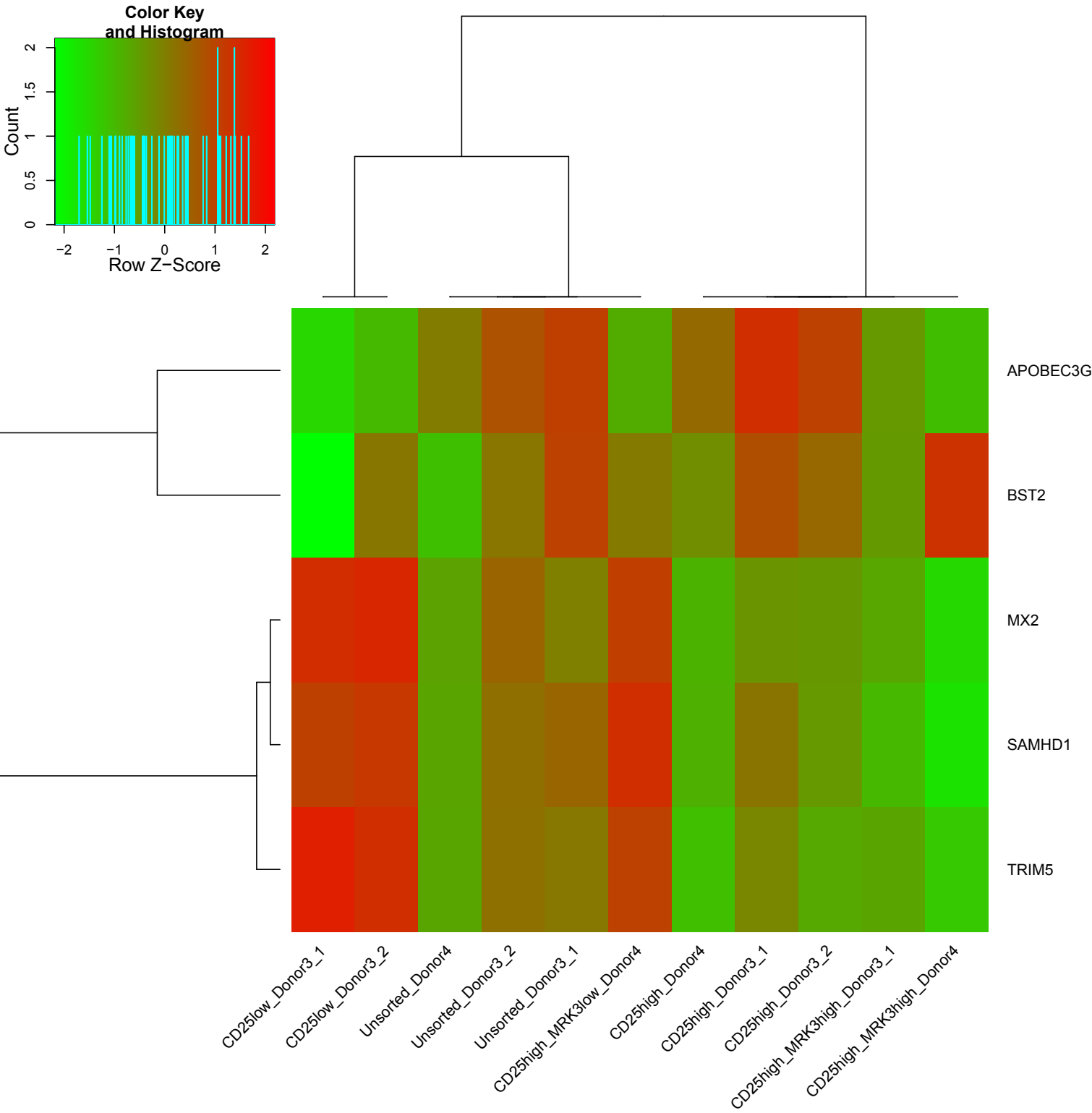

Supplement: S15 Fig — Complete hierarchical clustering of genes and cell samples was based on Pearson correlation. Color scale indicated in the legend corresponds to z-scores of gene expression levels expressed as the log10 of the number of library size-normalized reads per kilobase of exonic sequence, ranging from green (low) to red (high) expression. (PDF) [file ppat.1006678.s015.pdf]
